# Computational and Experimental IM-MS Determination of the Protonated Structures of Antimalarial Drugs

**DOI:** 10.1021/jasms.4c00207

**Published:** 2024-07-23

**Authors:** Younes Valadbeigi, Tim Causon

**Affiliations:** † Department of Chemistry, Faculty of Science, Imam Khomeini International University, Qazvin 34148-96818, Iran; ‡ BOKU University, Department of Chemistry, Institute of Analytical Chemistry, Muthgasse 18, Vienna 1190, Austria

## Abstract

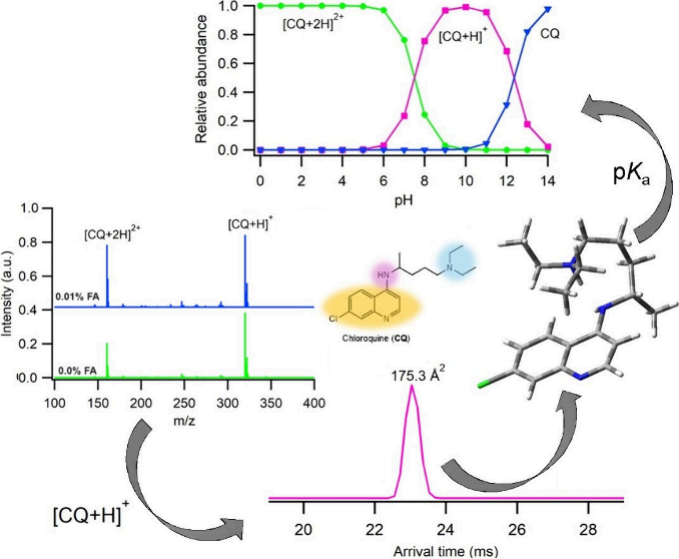

A combination of ion mobility-mass spectrometry (IM-MS) measurements and computational methods were used to study structural and physicochemical properties of a range of quinoline-based drugs: amodiaquine (AQ), cinchonine (CIN), chloroquine (CQ), mefloquine (MQ), pamaquine (PQ), primaquine (PR), quinacrine (QR), quinine (QN), and sitamaquine (SQ). In experimental studies, ionization of these compounds using atmospheric pressure chemical ionization (APCI) yields monoprotonated species in the gas phase while electrospray ionization (ESI) also produces diprotonated forms of AQ, CQ, and QR and also for PQ, SQ, and QN in the presence of formic acid as an additive. Comparison of the trajectory-method-calculated and experimental IM-derived collisional cross sections (CCS_*N2*_) were used to assign both the protonation sites and conformer geometry of all drugs considered with biases of 0.7–2.8% between calculated and experimental values. It was found that, in solution, AQ and QR are protonated at the ring nitrogen of the quinoline group, whereas the other drugs are protonated at the amine group of the alkyl chain. Finally, the conformers of [M + H]^+^ and [M + 2H]^2+^ assigned according to the lowest energies and CCS_*N2*_ calculations were used to calculate the p*K*_a_ values of the antimalarial drugs and the relative abundance of these ions at different pH values that provided validation of the computational and experimental IM-MS results.

## 1 Introduction

Malaria is an infectious and life-threatening disease caused by a parasite from the *Plasmodium* group and spread to humans through the bite of female *Anopheles* mosquitoes.^1−3^ According to a World Health Organization (WHO)^4^ report in 2022, malaria disease rates decreased from 2010 to 2014, but increased from 2015 to 2021. This trend has generated increasing demand for developing more effective antimalarial medicines and studying the action mechanism of currently used drugs.^5^ Different drug classes including quinoline-related compounds, antifolates, endoperoxides, and antimicrobials are used for treatment and prophylaxis of malaria.^6^ Quinine is the oldest effective antimalarial drug,^7^ but, because of some concerns about the toxicity, numerous quinoline derivatives including chloroquine, amodiaquine, and mefloquine were synthesized to replace quinine starting in the 1920s. Among these, chloroquine and primaquine became the most widely used during the 20th century for treatment against simpler forms of malaria.^8,9^ In addition to continued use for malaria treatment, some of these drugs have also been investigated for potential repurposing including most notably the combination of chloroquine alongside hydroxychloroquine as a treatment for COVID-19, which ultimately proved to have little to no impact on illness, hospitalization, or death.^10^

In the case of their conventional purpose, the antimalarial mechanism of quinoline derivatives is related to the prevention of the detoxification process in the *Plasmodium* parasites.^11,12^ When *Plasmodium* invades the cell, it digests the hemoglobin of red blood cells into a toxic complex, hematin, which can destroy *Plasmodium*. Hence, the parasite converts the toxic hematin to a nontoxic polymer, hemozoin.^13^ Although the precise action mechanism of the antimalarial drugs is not completely understood, two possible mechanisms have been suggested: (i) the antimalarial agents bind to hematin and prevent its conversion to hemozoin, and (ii) they accumulate in acidic food vacuoles of the malaria parasite, exerting antimalarial effects by increasing the pH.^14,15^ Based on both antimalarial mechanisms, it can be inferred that the structure and basicity (protonation extent) of the quinoline derivatives play a key role in the treatment and control of the symptoms of the *Plasmodium* parasite. In particular, the ionizable nitrogen heteroatom of quinoline is expected to be protonated in an acidic environment (e.g., inside the vacuoles of a parasite), while the additional 4-amino group present in many antimalarial drugs may also be protonated leading to an increased internal pH of lysosomes and induction of lysosomal mediated cellular toxicity and ultimately leading to cell cycle arrest. Hence, the investigation of interaction of these drugs, mainly chloroquine, with hematin has been the subject of several theoretical and experimental studies using different techniques including Raman spectroscopy,^16−20^ NMR,^21^ mass spectrometry (MS),^22^ and extended X-ray absorption fine structure (EXAFS) spectroscopy.^23,24^ These studies have revealed that the pH value and the protonation state of chloroquine can affect the interaction of chloroquine and hematin. The diprotonated chloroquine forms a noncovalent bond with hematin with the π–π structure,^16,25^ while the formation of an Fe–N bond has been reported between hematin and the neutral forms of chloroquine.^23^ Other than the interaction of these drugs with hematin, their transport into the cell is a crucial step in the treatment which is influenced by the structure and charge of the drugs. For example, it has been found that only the monoprotonated forms of mefloquine, quinine, and 7H-quinoline can cross the membrane of *Escherichia coli*.^26^ To date, there are only a few studies published and these have been limited to one drug^27−29^ or only one conformer of the neutral forms of some of the drugs.^30^ Due to the sustained use of most of these drugs for malaria treatment in single or combined dosages, as well as continuing investigations of their potential repurposing, analytical methods based on mass spectrometry for quantification of these drugs, their metabolites and degradation products have been the focus of several analytical studies for which electrospray (ESI) in the positive mode is the preferred method of ionization.^31−33^

In this regard, it is notable that quinoline drugs used for malaria treatment possess two or three basic sites, meaning that their monoprotonation can produce a mixture of isomeric protonated ions, so-called “protomers” in the case of positive mode ionization. Furthermore, because the alkyl chain present in their structures can provide additional flexibility, all neutral, mono- and diprotonated forms of these drugs may take the form of several different conformers that can be studied with ion mobility-mass spectrometry (IM-MS), which has already been proven as a valuable approach for studying various related drugs including the assessment of their physicochemical properties.^34,35^ Separation of protomers and conformers of ions with IM is possible due to differences in their collision cross section (CCS).^36^ Hence, IM-MS in combination with computational methods has been used in chemistry, biophysics, and biology to study the structure of biomolecules and small molecules^37,38^ and to determine the protonation sites in compounds with more than one proton acceptor site.^39,40^ Furthermore, as the ionization of compounds in ESI-IM-MS is based on protonation/deprotonation, this technique can be used to estimate the p*K*_a_ values of different compounds.^41,42^ In this work, the protonation of nine quinoline-based antimalarial drugs is experimentally studied by IM-MS using ESI and atmospheric pressure chemical ionization (APCI) ion sources. The site of protonation and the most probable conformers of the protonated forms of the drugs are determined using the IM-derived collisional cross sections (^*DT*^CCS_N2_) and computational methods with the aim of obtaining correct p*K*_a_ values to predict the relative abundances of mono- and diprotonated forms of these drugs at different pH values.

## 2 Experimental Section

### 2.1 Instrumentation

An Agilent 6560 IM-QTOF mass spectrometer was used for all measurements. A Dual Jetstream electrospray ion source (ESI) and a corona discharge atmospheric pressure chemical ionization (APCI) source (G1947B, Agilent Technologies) were used for ionization of the compounds. The instrument was calibrated prior to measurements in the 2 GHz extended dynamic range mode using the standard Dual Jetstream ESI ion source and following the recommended tune procedure of the manufacturer. The temperature and flow rate of the drying gas in ESI were 225 °C and 13 L min^–1^, respectively. The temperature of the vaporizer and pressure of the nebulizer in APCI were 350 °C and 30 psi, respectively. The drift tube was operated with a pressure of 3.94 Torr at 26–27.25 °C with high purity nitrogen as the drift gas (Linde Gas GmbH, Vienna). A trap release time of 150 μs, trap filling time of 10 ms, and maximum arrival time of 60 ms were applied as standard settings for IM-MS measurements, while a trap filling time of 1.25 ms was used for acquisition with 4-bit multiplexing. Targeted collision induced dissociation (CID) measurements were performed using the “narrow” precursor *m*/*z* selection (width of ∼1.3 u) and fixed collision energies using nitrogen as collision gas.

### 2.2 Materials and Methods

Amodiaquine dihydrochloride (>99%), cinchonine (98%), chloroquine disulfate (98%), mefloquine hydrochloride (98.1%), pamaquine naphthoate (Aldrich^CPR^), primaquine bisphosphate (98%), quinacrine dihydrochloride (90%), quinine hemisulfate salt monohydrate (>98%), and sitamaquine tosylate (98%) were purchased from Sigma-Aldrich (Vienna, Austria). Formic acid (97.5%) was purchased from Honeywell. Standard solutions with a concentration of 10 μmol L^–1^ were prepared in deionized water. A syringe pump (KD Scientific, Series 100, USA) was used to infuse solutions with flow rate of 20 μL min^–1^ into the nebulizer. A commercially available tune mix (ESI-L Low Concentration Tuning Mix, G1969–85000, Agilent Technologies) was prepared according to manufacturer instructions for tuning and accurate mass calibration of the mass spectrometer. Agilent IM-MS Browser 10.0 was used for single-field calibration and ^*DT*^CCS_N2_ calculation. PNNL Preprocessor was used for demultiplexing and data preprocessing steps,^43^ Agilent Mass Profiler 10.0.2.202 was used for feature extraction, followed by high-resolution demultiplexing (HRdm) of the DTIM-MS data with Agilent HRdm 2.0.^44^

### 2.3 Computational Details

Structures of all neutral, mono- and diprotonated forms of the antimalarial drugs were fully optimized by density functional theory (DFT) with ωB97xD functional. The basis set 6-311++G(d,p) including diffuse and polarization functions for hydrogen and heavy atoms was used for the calculations. Conformational search was performed by GaussView 6.1.1 software and the GMMX add-on module using the MMFF94 force field. Charge distribution calculation was carried using the Merz–Kollman (MK) method at the same level of theory. Tomasi’s Polarized Continuum Model (PCM)^45^ was used for calculations in water solvent. The p*K*_a_ values for mono- and diprotonation of the compounds were calculated using eq 1
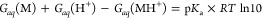
1where MH^+^ is the protonated molecule and M is its conjugated base, *R* is the gas constant, and *T* is the temperature. *G*_*aq*_(M), *G*_*aq*_(MH^+^), and *G*_*aq*_(H^+^) are the respective Gibbs energies of the conjugate base (M), the protonated molecule (MH^+^), and the proton (H^+^) in solution. *G*_*aq*_(H^+^) was considered as 265.6 kcal mol^–1^ (1111.27 kJ mol^–1^)^46^ for the p*K*_a_ calculations. Gaussian 16 software^47^ was used for DFT calculations. The DFT output files containing geometric parameters of the optimized structures of (di)-protonated ions and MK charges were used to build the input files for collision cross section (CCS) calculations. CCS calculations were performed by MOBCAL-MPI software^48^ using the trajectory method (TM) in nitrogen as buffer gas and at 298 K.

## 3 Results and Discussion

### 3.1 Ionization Mechanism of the Antimalarial Drugs in ESI and APCI Ion Sources

**Figure 1:**
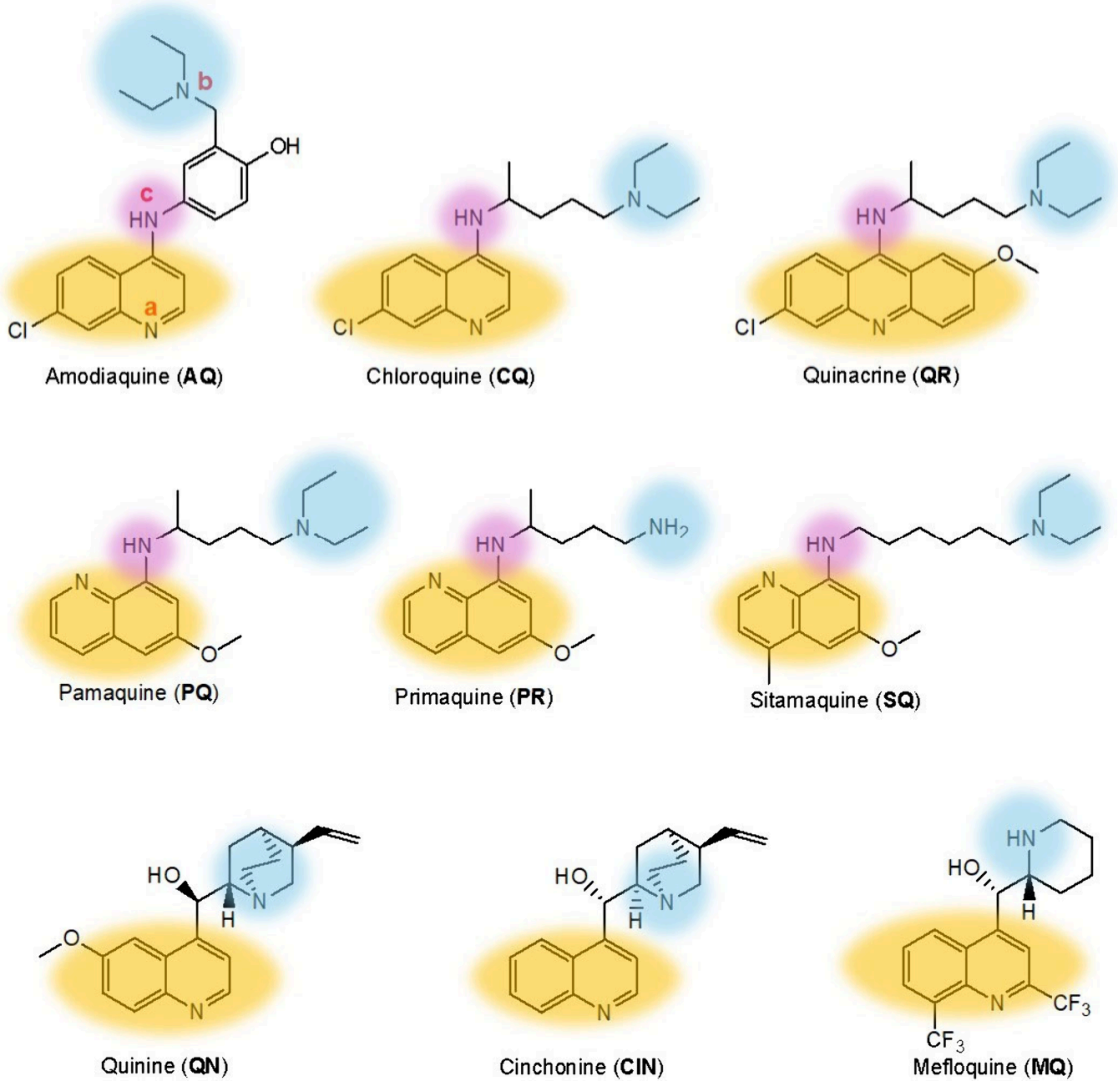
Chemical structures of the antimalarial drugs amodiaquine (AQ), cinchonine (CIN), chloroquine (CQ), mefloquine (MQ), pamaquine (PQ), primaquine (PR), quinacrine (QR), quinine (QN), and sitamaquine (SQ). Different proton acceptor sites including the nitrogen atom of the quinoline ring (yellow), the nitrogen atom of the alkyl group (blue), and the nitrogen atom directly connected to the ring (purple) are indicated by letters **a**, **b**, and **c**, respectively.

Figure 1 shows the structures of nine antimalarial drugs studied in this work: amodiaquine (AQ), cinchonine (CIN), chloroquine (CQ), mefloquine (MQ), pamaquine (PQ), primaquine (PR), quinacrine (QR), quinine (QN), and sitamaquine (SQ). Although all of these compounds are quinoline derivatives, they can be further classified into three structural groups: (i) AQ, CQ, and QR which are derivatives of 1,4-aminoquinoline, (ii) PQ, PR, and SQ with 1,8-aminoquinolne scaffolds, and (iii) QN, CIN, and MQ which lack a secondary amine at the 4-position of the quinoline. The compounds with 1,4- and 1,8-aminoquinoline scaffolds thus possess three nitrogen atoms that can act as proton acceptor sites indicated by **a**, **b**, and **c** in Figure 1, while QN, CIN, and MQ have only two proton acceptor sites at N-**a** (pyridine) and N-**b** (amine nitrogen). The optimized structures of different conformers of the neutral forms of the antimalarial drugs are provided in Figures S1–S9 (see Supporting Information). In addition, the relative Gibbs free energies of the conformers in the gas phase and in solutions are compared in Figures S1–S9. Of note is that, for compounds possessing a long alkyl chain (CQ, QR, PQ, SQ), the most stable conformers are those with a compact structure in which the alkyl chain is bent on the aromatic rings. Although from the entropy point of view, these compact structures are less favored than the extended structures, internal interactions (NH···N hydrogen bond in CQ and CH···π interaction in QR, PQ, and SQ) stabilize the conformers with the compact structures. The most stable conformers of AQ, QN, CIN, and MQ are those with an intramolecular OH···N hydrogen bond.

**Figure 2:**
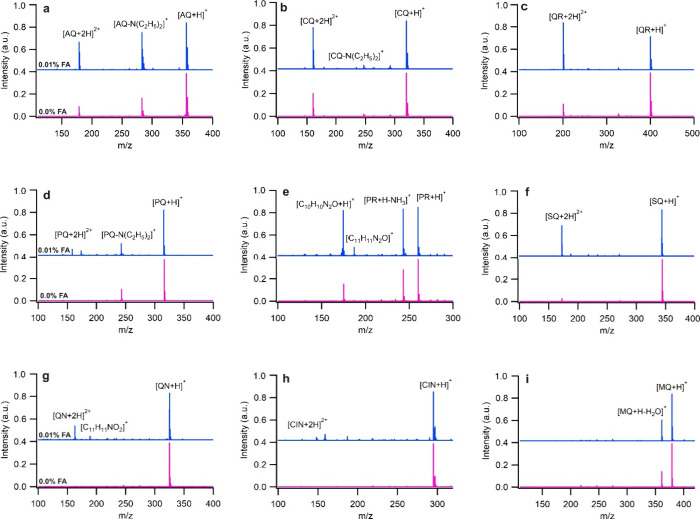
ESI mass spectra of (a) AQ, (b) CQ, (c) QR, (d) PQ, (e) PR, (f) SQ, (g) QN, (h) CIN, and (i) MQ in aqueous solvent without and with 0.01% FA.

The experimental ESI mass spectra of the antimalarial drugs in aqueous solution including the effect of formic acid (FA) additive (0.01%) are shown in Figure 2. In all mass spectra, an intense peak is observed for [M + H]^+^ irrespective of the presence or absence of FA as an additive. However, only the 1,4-aminoquinoline derivatives (AQ, CQ, and QR) are observed to yield significant quantities of [M + 2H]^2+^ in the absence of FA indicating that diprotonation of the antimalarial drugs depends on their structures. Computational results reveal that protonation of the pyridine nitrogen atom (site N-**a**) of both 1,4- and 1,8-aminoquinoline leads to delocalization of the positive charge of the entering proton via resonance structures illustrated in Figure S10. However, in the case of 1,8-aminoquinoline, the positive charge is only delocalized on the aromatic rings while, in the case of 1,4-aminoquinoline, the amine group can also participate in charge delocalization. Although the more charge delocalization in 1,4-aminoquinoline derivatives does not lead to a significant increase in the basicity of their N-**a** atom compared to basicity of the corresponding N-**a** atom of 1,8-aminoquinoline derivatives (Δ*H* of monoprotonation in Table 1), charge delocalization stabilizes the monoprotonated compound and prepares it for accommodating an additional positive charge. This is observed in the Δ*H* of diprotonation of the 1,4-aminoquinoline derivatives (AQ, CQ, and QR) which are meaningfully larger than Δ*H* of diprotonation of the 1,8-aminoquinoline derivatives apart from SQ. SQ has the longest alkyl chain of the studied drugs with six carbon atoms enabling the maximum separation between the positive charge centers in [SQ+2H]^2+^.

**Table 1:** Calculated Δ*H* and Δ*G* for Mono- and Di-Protonation of the Antimalarial Drugs in the Gas Phase and Their p*K*_a1_ and p*K*_a2_ Values in Water Solvent^a^

	Gas phase monoprotonation		Gas phase diprotonation^c^		p*K*_a_ in solution^d^	
Compound	Δ*H* (kJ mol^–1^)	Δ*G* (kJ mol^–1^)	Δ*H* (kJ mol^–1^)	Δ*G* (kJ mol^–1^)	p*K*_a1_	p*K*_a2_
AQ (**a**)^b^	–1029.3	–999.7	–1816.7	–1754.2	6.21	10.27
AQ (**b**)	–996.3	–957.1				
AQ (**c**)	–895.9	–863.3				
CQ (**a**)	–1020.3	–994.1	–1807.9	–1755.7	7.51 (7.29)^e^	12.34 (10.32)^e^
CQ (**b**)	–1023.3	–987.8				
CQ (**c**)	–904.8	–874.1				
QR (**a**)	–1074.6	–1033.9	–1827.9	–1767.8	8.78 (8.37)^e^	12.79 (10.33)^e^
QR (**b**)	–1034.6	–996.1				
QR (**c**)	–995.2	–954.8				
PQ (**a**)	–1021.0	–984.6	–1791.2	–1735.2	2.03	12.54
PQ (**b**)	–1070.6	–1036.4				
PQ (**c**)	–973.2	–944.3				
PR (**a**)	–1017.8	–986.5	–1721.1	–1668.5	0.23	10.69
PR (**b**)	–1025.1	–989.5				
PR (**c**)	–1015.1	–980.4				
SQ (**a**)	–1020.4	–983.0	–1831.7	–1779.6	3.11	12.03
SQ (**b**)	–1060.0	–1025.0				
SQ (**c**)	–1033.7	–998.0				
QN (**a**)	–1000.2	–968.5	–1756.9	–1694.7	3.46	10.63
QN (**b**)	–1010.1	–978.3				
CIN (**a**)	–993.3	–961.0	–1746.5	–1686.0	3.33	9.74
CIN (**b**)	–1008.7	–975.8				
MQ (**a**)	–931.1	–898.8	–1632.7	–1570.4	–7.31	7.96
MQ (**b**)	–957.4	–927.6				

a The most stable protomers and conformers have been considered for the calculations in each phase.

b The letters in parentheses indicate the site of monoprotonation (see Figure 1).

c For diprotonation, the most basic sites **a** and **b** are protonated.

d *pK*_*a1*_ and *pK*_*a2*_ have been computed for deprotonation of [M + 2H]^2+^ and [M + H]^+^, respectively.

e From ref (50).

Although the structure is a determining factor in the protonation of the antimalarial drugs, other factors including solvent and pH should not be neglected as they may have even more effect on the degree of protonation. To investigate effect of solvent on protonation, ionization of the antimalarial drugs in the gas phase was studied using an APCI ion source where the solvent effect is absent. Figure S11 shows the mass spectra of the antimalarial drugs ionized with the APCI ion source with and without FA as an additive. In APCI, only monoprotonated ions, [M + H]^+^, are formed and no peak was observed for [M + 2H]^2+^. In addition, the presence of FA has no discernible effect on the degree of protonation nor on the overall mass spectra, which is in expected stark contrast to the ESI results. Additional measurements using ESI with 0.01% FA confirm that FA has a considerable effect on the diprotonation of the drugs (Figure 2). In the presence of FA, the intensity of the [M + 2H]^2+^ peak of AQ, CQ, and QR increases and a new peak for the diprotonation of PQ, SQ, and QN appears while PR and MQ are not diprotonated even with 0.01% FA as additive.

Other than the dominant [M + H]^+^ and [M + 2H]^2+^ ions, some in-source fragment peaks are observed in the ESI mass spectra of the antimalarial drugs that provide some insight into their gas-phase ion structures. The measured *m*/*z* of the fragment ions and their formula are provided in Table S1. The peaks with *m*/*z* of 283.0632, 247.1008, and 243.1503 in the mass spectra of AQ, CQ, and PQ correspond to the loss of NH(C_2_H_5_)_2_ upon protonation. Other than NH_3_ loss (*m*/*z* 243.1491), the mass spectrum of PR also contains two fragment peaks with *m*/*z* of 175.0857 ([C_10_H_11_N_2_O]^+^) and 187.0862 ([C_11_H_11_N_2_O]^+^) which are due to the loss of the alkyl amine chain. The fragment [C_10_H_11_N_2_O]^+^ is the protonated form of 8-amino-6-methoxyquinoline, and [C_11_H_11_N_2_O]^+^ contains an additional C atom connected to the aniline nitrogen with a double bond, −NH^+^=CH_2_ (Table S1). The observed fragmentation patterns from experimental ESI and APCI mass spectra are similar. However, in the cases of CQ and QR, additional fragments are observed in APCI corresponding to N(C_2_H_5_)_2_ loss followed by hydride abstraction from the alkyl chain and formation of [CQ-N(C_2_H_5_)_2_-2H]^+^ and [QR-N(C_2_H_5_)_2_-4H]^+^ ions, respectively. Hydride abstraction in this type of APCI source occurs via reaction of the compounds with reactant ion NO^+^.^49^

### 3.2 Conformational Analysis and Protonation Sites of [M + H]^+^ and [M + 2H]^2+^ Ions

Having established a primary experimental data set for all drugs, computational methods were then used to discern all possible protomers (protonated isomers) and conformers corresponding to each ^*DT*^CCS_N2_ value and evidence the protonation site of each antimalarial drug and its observed gas-phase structure. For each molecule, protonation at all relevant basic sites (see N-**a**, N-**b**, and N-**c** in Figure 1) was considered in the conformational searching. For the compounds with an alkyl chain (CQ, QR, PQ, PR, SQ), the 10 most stable conformers were selected while fewer numbers of conformers were obtained for the other molecules with more rigid structures (QN, CIN, MQ). Subsequently, the geometries of the selected conformers were fully optimized in the gas phase and in solution to make a comprehensive comparison. Some conformers converged upon geometry optimization, hence, only one of them was kept for the CCS_*N2*_ calculations. The optimized structures of all conformers and protomers of the monoprotonated forms of the antimalarial drugs are provided in Figures S12–S20, and their Gibbs energies in the gas phase and solution, their relative abundances in solution, and their calculated CCS_*N2*_ values are summarized in Table S2–S10.

**Figure 3:**
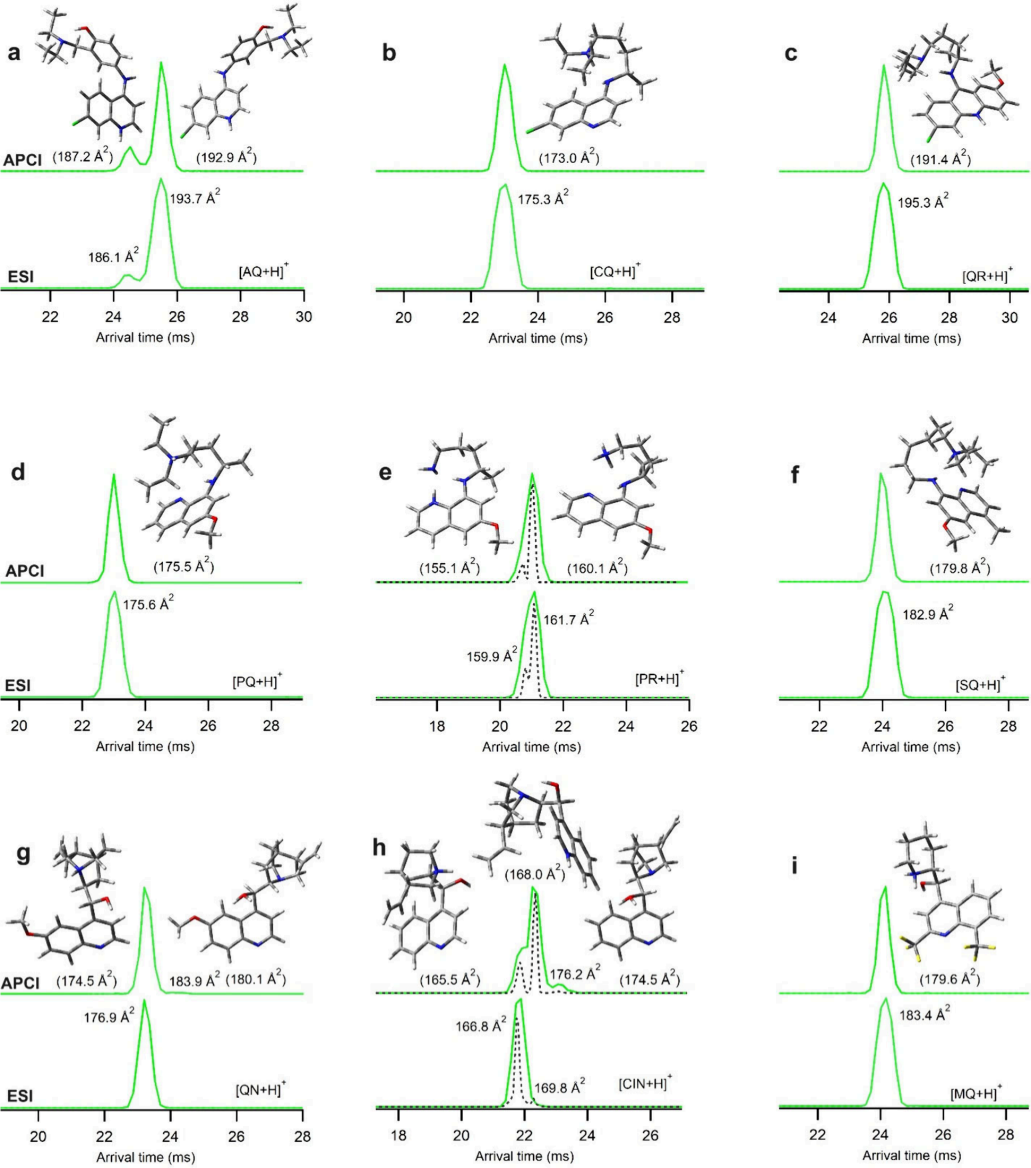
Comparison of the IM spectra of [M + H]^+^ ion of (a) AQ, (b) CQ, (c) QR, (d) PQ, (e) PR, (f) SQ, (g) QN, (h) CIN, and (i) MQ produced by APCI and ESI ion sources. The dashed lines are the corresponding IM spectra obtained by high resolution demultiplexing (HRdm) for PR and CIN. HRdm was done for IM spectra of other compounds and no additional peak was observed. The calculated CCS_*N2*_ values are provided in parentheses.

Figure 3 compares the *m*/*z*-selected ion mobility (IM) spectra of the monoprotonated forms ([M + H]^+^) of the antimalarial drugs produced by APCI and ESI ion sources in aqueous solution. The experimentally derived ^*DT*^CCS_N2_ value (Å^2^) for each peak is also shown. Both the IM spectra of AQ obtained by APCI and ESI reveal two peaks with the experimental ^*DT*^CCS_N2_ values of 186.1 and 193.7 Å^2^. These peaks may represent either two protomers or two conformers of one protomer. Comparison of the relative Gibbs energies of the monoprotonated structures of AQ shows that N-**c** is the weakest base both in the gas phase and in solution, while the nitrogen of the quinoline group (N-**a**) is more basic than N-**b** by about 50 and 20 kJ mol^–1^, in the gas phase and in solution, respectively (Table S2). Hence, the more intense IM peak with ^*DT*^CCS_N2_ value of 193.7 Å^2^ is rationalized to be the most stable conformer of AQ protonated at N-**a** with the calculated CCS_*N2*_ of 192.9 Å^2^ (Figure 3a). The small peak with ^*DT*^CCS_N2_ value of 186.1 Å^2^ might be attributed to protonation at amine nitrogen N-**b**, but this nitrogen is a weaker base than N-**a** and no conformer of this protomer matches the ^*DT*^CCS_N2_ of this peak. Hence, the small peak seems to be an additional N-**a**-protonated conformer with the calculated CCS_*N2*_ of 187.2 Å^2^ (Figure 3a). The IM spectra of [CQ + H]^+^ and [QR + H]^+^ show only one peak with both APCI and ESI. As N-**b** is a stronger base than N-**a** in solution for CQ, it is rationalized to be protonated at the N-**b** amine group to produce a stable conformer with the calculated CCS_*N2*_ of 173.0 Å^2^ providing the best agreement with the experimental ^*DT*^CCS_N2_ value of 175.3 Å^2^ (Figure 3b). The N-**a** site of QR is more basic than its N-**b** site both in the gas phase and in solution (Table S4). The calculated CCS_*N2*_ of the most stable conformer of this N-**a** protomer is 191.4 Å^2^ which provides a reasonable match to the observed ^*DT*^CCS_N2_ value of 195.3 Å^2^. There is also another stable conformer for QR protonated at N-**b** with the calculated CCS_*N2*_ of 195.0 Å^2^, but it is less stable than the N-**a** protomer by 5.9 and 55 kJ mol^–1^ in solution and in the gas phase, respectively (Table S4). The IM spectra of the 1,8-aminoquinolne derivatives [PQ + H]^+^ and [SQ + H]^+^ reveal only one peak both in ESI and APCI (Figure 3d and f). For these compounds, the N-**b** atom is the most basic site both in the gas phase and in solution (Tables S5–S7). The calculated CCS_*N2*_ of the most stable conformers of [PQ + H]^+^ and [SQ + H]^+^ protonated at N-**b** are 175.5 and 179.8 Å^2^, respectively, showing good agreement with the corresponding experimental ^*DT*^CCS_N2_ values of 175.6 and 182.9 Å^2^. The IM spectra of [PR + H]^+^ with ESI and APCI show an intense peak with an experimental ^*DT*^CCS_N2_ of 160.1 Å^2^ in standard mode corresponding to the most stable conformer protonated at N-**b**. However, the HRdm-evaluated^37^ IM spectrum of PR reveals a small peak with an experimental ^*DT*^CCS_N2_ of 159.1 Å^2^ which is likely to be the most stable conformer of [PR + H]^+^ protonated at N-**a** with the calculated CCS_*N2*_ of 155.1 Å^2^. In contrast, QN and CIN with similar structures show one dominant IM peak for their monoprotonation by ESI (Figure 3g,h) with experimental ^*DT*^CCS_N2_ values of 176.9 and 166.8 Å^2^, respectively. Regarding the relative protonation energies in solution (Tables S8 and S9), N-**b** is the most basic site of QN and CIN; hence, the observed major IM peaks in ESI are attributed to protonation of N-**b** protomers of QN and CIN with calculated CCS_*N2*_ values of 174.5 and 165.5 Å^2^, respectively. In APCI, an additional intense peak for CIN with the ^*DT*^CCS_N2_ of 169.8 Å^2^ is observed which is due to protonation of its N-**a** atom (only observed in trace amounts for ESI). A corresponding additional IM peak is not observed for QN with the APCI ion source, probably because the CCS_*N2*_ values of both N-**a** and N-**b** protomers of QN are similar (with calculated CCS_*N2*_ values of 174.8 and 174.5 Å^2^, respectively) and their IM peaks overlap. The two additional small peaks with the experimental ^*DT*^CCS_N2_ values of 183.9 and 176.2 Å^2^ are probably due to protonation of the OH groups of QN and CIN in the gas phase with the calculated CCS_*N2*_ values of 180.1 and 174.5 Å^2^, respectively. Although we did not observe the [M + H – H_2_O]^+^ fragment for water elimination in the APCI mass spectra of QN and CIN (Figure S11), which is the characteristic peak confirming protonation of OH group, we could observe this fragment using CID measurement with a CID voltage of 10 V (Figure S21). Only one IM peak was observed for MQ with both APCI and ESI which is attributed to protonation of its amine group (N-**b**) as this site is a stronger base than the N-**a** site by about 40 and 80 kJ mol^–1^ in the gas phase and in solution, respectively (Table S10). In summary, as the protonation in the gas phase is fast, a greater number of basic sites can be protonated in APCI regardless of their basicity (kinetic controlled protonation). However, in solution, the antimalarial drugs have enough time and only their most basic site is protonated in ESI (thermodynamic controlled protonation).

Structural analysis of the diprotonated forms is relatively simpler as their protonation sites have been already determined (i.e., protonation of both N-**a** and N-**b**) and only the correct conformer for each ion needs to be identified. The optimized structures of the conformers of diprotonated forms of the antimalarial drugs are provided in Figures S22–S30 and their Gibbs energies in the gas phase and solution and their calculated CCS_*N2*_ values are summarized in Tables S11–S19. The *m*/*z*-selected IM spectra of [AQ+2H]^2+^, [CQ+2H]^2+^, [QR+2H]^2+^, [PQ+2H]^2+^, [SQ+2H]^2+^, and [QN+2H]^2+^ are shown in Figure S31 illustrating that only one IM peak was observed for each ion. The most stable conformer and its calculated CCS_*N2*_ value are also provided in Figure S31. In comparison to the monoprotonated forms, whereby the folded structures are the most stable conformers, the diprotonated ions are extended structures that maximize the distance between the two protonation sites as illustrated by the increases of 22–37% in the experimental ^*DT*^CCS_N2_ compared to the monoprotonated ions (Figure S31).

As the antimalarial drugs function in the body (aqueous solution), we present only the results of ESI in the aqueous solution to obtain the most realistic insight into the physicochemical properties of these drugs. However, the ESI measurements were repeated in a CH_3_OH:H_2_O (90:10) solution and the same ion mobility spectra were observed (Figure S32).

### 3.3 Determination of p*K*_a_ Values and Relative Abundances of [M + H]^+^ and [M + 2H]^2+^ in Aqueous Solution

**Figure 4:**
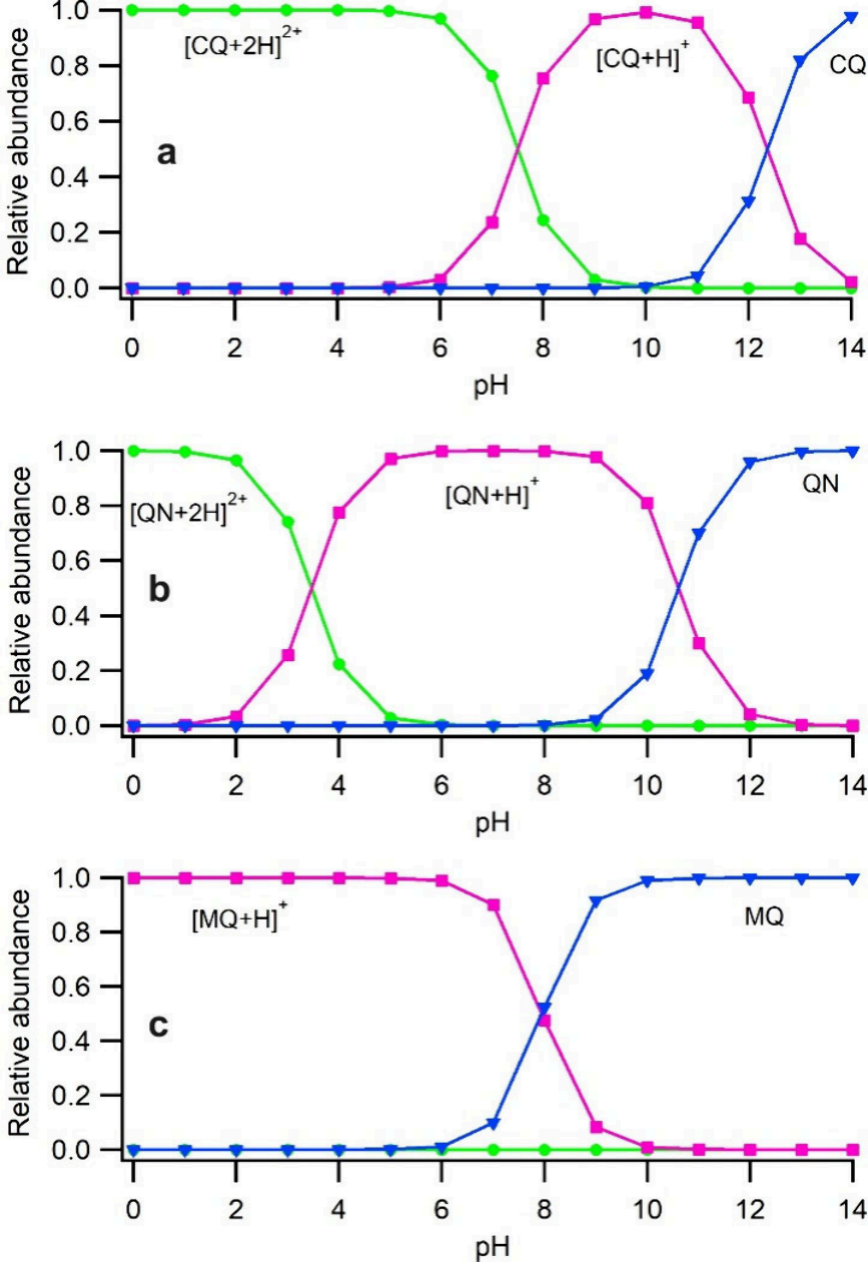
Calculated relative abundances of the [M + 2H]^2+^, [M + H]^+^, and M species of (a) CQ, (b) QN, and (c) MQ at different pH values in the aqueous solution.

Based on the protonation state, the structures of antimalarial drugs considered in this work can be categorized into lipophilic form (LP), cationic amphiphilic drug (CAD), and hydrophilic form (HP) corresponding to M, [M + H]^+^, and [M + 2H]^2+^ forms, respectively.^50^ The abundance of the LP, CAD, and HP structures in solution is determined by their p*K*_a1_ and p*K*_a2_ values representing the deprotonation of [M + 2H]^2+^ and [M + H]^+^, respectively. Using the structures determined based on the experimental and theoretical CCS_*N2*_ data in the present work, the p*K*_a1_ and p*K*_a2_ values for the antimalarial drugs were calculated and compared with the available reported p*K*_a_ values in Table 1. Although our p*K*_a1_ values are in good agreement with those reported in ref (50), the p*K*_a2_ calculated in this work are larger than those in ref (50) by about 2 units. This difference may originate from two sources: (i) the methods used for calculations of the p*K*_a_ are essentially different and (ii) the previous study considered the amine group (N-**b**) of QR as the protonation site in [QR + H]^+^ while we determined the ring nitrogen (N-**a**) is the correct protonation site. To confirm the accuracy of the calculated p*K*_a_ values in the present work, we used these values to calculate the relative abundances of the M, [M + H]^+^, and [M + 2H]^2+^ and then compared results for [M + H]^+^ and [M + 2H]^2+^ with the relative peak intensities of these ions in the ESI mass spectra in Figure 2. For the p*K*_a_ calculations, the most stable conformer in solution was selected, which was the same in gas phase and solution for all examples except in the cases of [QN + H]^+^ and [CQ + H]^+^. In the case of [QN + H]^+^ the most stable structures in the gas phase and in solution are two conformers of the same protomer protonated at N-**b** (aliphatic nitrogen). In contrast, the most stable isomers of [CQ + H]^+^ in the gas phase and in the solution are two protomers protonated at N-**a** (ring nitrogen) and N-**b** (alkyl nitrogen), respectively. The most stable isomers of [QN + H]^+^ and [CQ + H]^+^ in the gas phase are less stable than the most stable isomers in solution by 10 and 12 kJ mol^–1^, respectively. Using the most stable conformers of [QN + H]^+^ and [CQ + H]^+^ in the gas phase for the p*K*_a_ calculations leads to a shift of almost 2 units in both p*K*_a1_ and p*K*_a2_ which predicts that QN can be slightly diprotonated in the absence of FA which is not in agreement with the mass spectrometry results. The plots of relative abundances of M, [M + H]^+^, and [M + 2H]^2+^ of all antimalarial drugs at different pH are provided in Figure S33. In Figure 4, these results for CQ, QN, and MQ that exhibited distinctly different distributions of these ion species in the ESI mass spectra are highlighted. Figure 2 shows that CQ produces both [CQ + H]^+^ and [CQ+2H]^2+^ with and without the presence of FA. Conversely, QN is only monoprotonated in the absence of FA, but it produces a small amount of [QN+2H]^2+^ in the presence of FA, and MQ only produces [MQ + H]^+^ with no [MQ+2H]^2+^ peak observed irrespective of the presence of FA. The calculated relative abundances in Figure 4 are qualitatively in accordance with our experimental observations indicating that the calculated p*K*_a1_ and p*K*_a2_ values for the antimalarial drugs are reliable data.

## 4 Conclusions

In this work, we showed that experimental measurements with ESI-IM-MS can provide qualitative information on the protonation state (mono- and diprotonation) of several quinoline derivative drugs in solution. The possible conformers and protomers of each compound have a broad range of structures and energies leading to a range of possible physicochemical properties including p*K*_*a*_ for each compound. To confirm a single reliable *pK*_a_ value, determination of the correct protomers and conformers in solution is crucial; thus, computational methods in combination with the IM-derived CCS_*N2*_ data provide a means to confirm the protonation site, and the correct conformer of protomers in the gas phase and solution. Structurally, it could be shown that the position of secondary amine (N-**c**) on the quinoline ring influences the preferred site of protonation and also the degree of diprotonation as exhibited for the 1,4-aminoquinoline derivative drugs which were preferably protonated at the ring nitrogen and exhibited a higher degree of diprotonation compared to the 1,8-aminoquinoline derivatives. Beyond the results shown in this study, the methods used in this work could be extended to further studies of quinoline-based drugs and their metabolites to determine their physicochemical properties and rationalize their ionization behavior for improving analytical methods to determine them.

## Supporting Information Available

Optimized structures and relative Gibbs energies of neutral AQ, CQ, CIN, MQ, PQ, PR, QR, QN, and SQ; resonance structures for protonated forms of aminoquinolines; APCI mass spectra; measured *m*/*z* and ion formula; optimized structures of conformers and protomers of [AQ + H]^+^, [CQ + H]^+^, [CIN + H]^+^, [MQ + H]^+^, [PQ + H]^+^, [PR + H]^+^, [QR + H]^+^, [QN + H]^+^, and [SQ + H]^+^; relative energies and calculated CCS_N2_ values of [AQ + H]^+^, [CQ + H]^+^, [CIN + H]^+^, [MQ + H]^+^, [PQ + H]^+^, [PR + H]^+^ [QR + H]^+^, [QN + H]^+^ and [SQ + H]^+^; APCI-mass spectra of [CIN + H]^+^ and [QN + H]^+^ with CID voltages of 10 and 20 V; optimized structures of conformers and protomers of [AQ+2H]^2+^, [CQ+2H]^2+^, [CIN+2H]^2+^, [MQ+2H]^2+^, [PQ+2H]^2+^, [PR+2H]^2+^, [QR+2H]^2+^, [QN+2H]^2+^, and [SQ+2H]^2+^; relative energies and calculated CCS_N2_ values of [AQ+2H]^2+^, [CQ+2H]^2+^, [CIN+2H]^2+^, [MQ+2H]^2+^, [PQ+2H]^2+^, [PR+2H]^2+^, [QR+2H]^2+^, [QN+2H]^2+^, and [SQ+2H]^2+^; ion mobility spectra of the [M + 2H]^2+^ ions; relative abundances of the [M + 2H]^2+^, [M + H]^+^, ion mobility spectra of [M + H]^+^ ions in water and methanol; and M species at different pH (PDF)
