# Supplementary material for: Computational and Experimental IM-MS Determination of the Protonated Structures of Antimalarial Drugs
Source: J Am Soc Mass Spectrom. 2024 Jul 23;35(8):1930–9. doi: 10.1021/jasms.4c00207 (PMC11311536; doi:10.1021/jasms.4c00207)
Supplement: Supplementary file 1 — js4c00207_si_001.pdf [file js4c00207_si_001.pdf]

Supporting Information

Computational and Experimental IM-MS Determination of the Protonated Structures of Antimalarial Drugs

Younes Valadbeigi*[a], Tim Causon*[b]

[a] Department of Chemistry, Faculty of Science, Imam Khomeini International University, Qazvin, 34148-96818, Iran; Email: valadbeigi@sci.iku.ac.ir.

[b] University of Natural Resources and Life Sciences, Vienna, Department of Chemistry, Institute of Analytical Chemistry, Muthgasse 18, Vienna, 1190, Austria; Email: tim.causon@boku.ac.at.

content	page
**Figures S1-S9**. Optimized and relative Gibbs energies of neutral antimalarial drugs	S2-S4
**Figure S10**. Resonance structures for protonated forms of aminoquinolines	S5
**Figure S11**. APCI mass spectra of the antimalarial drugs	S6
**Table S1**. The measured *m/z* and the ion formula	S7
**Figures S12-20**. Optimized structures of conformers of the $[M+H]^+$ ions	S8-S24
**Tables S2-S10**. Relative energies and calculated CCS_{N2} values of the $[M+H]^+$ ions	S9-S25
**Figure S21**. Mass spectra of $[ClN+H]^+$ and $[QN+H]^+$ with CID voltages of 10 and 20 V	S26
**Figures S22-S30**. Optimized structures of conformers of the $[M+2H]^{2+}$ ions	S27-S35
**Tables S11-S19.** Relative energies and calculated CCS_{N2} values of the $[M+2H]^{2+}$ ions	S27-S35
**Figure S31.** Ion mobility spectra of the $[M+2H]^{2+}$ ions	S36
**Figure S32**. Comparison of the ESI-ion mobility spectra of $[M+H]^+$ ions in water and methanol solvents	S37
**Figure S33.** Relative abundances of $[M+2H]^{2+}$, $[M+H]^+$, and M at different pH	S38

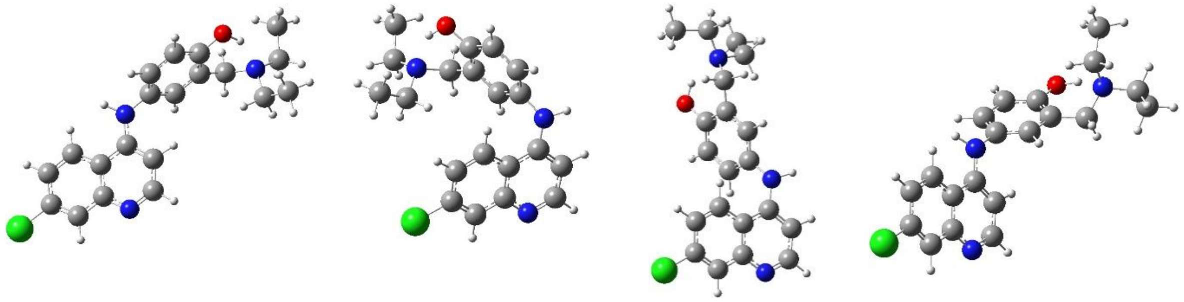

AQ-a 1.3 (0.0) | **AQ-b** 5.8 (5.3) | **AQ-c** 11.8 (9.9) | **AQ-d** 0.0 (0.8)

Figure S1. Optimized structures and relative Gibbs free energies of different conformers of neutral Amodiaquine (AQ) and in the gas phase and in aqueous solution (the numbers in the parenthesis). Relative energies are in kJ mol^{-1}.

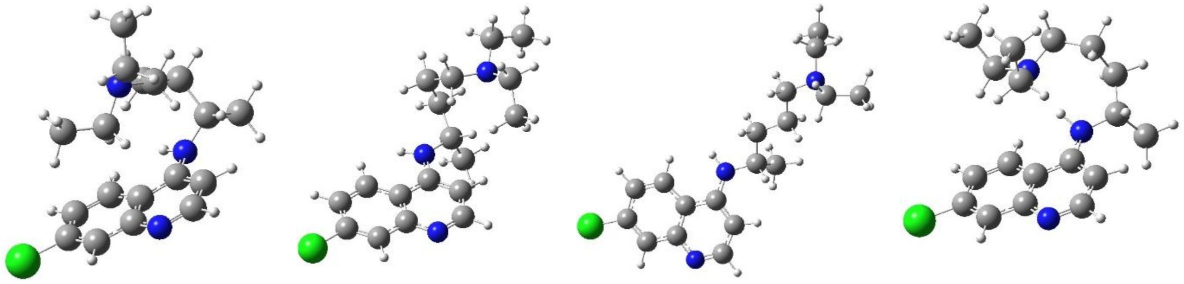

CQ-a 6.8 (4.2) | **CQ-b** 1.8 (0.0) | **CQ-c** 12.1 (6.7) | **CQ-d** 0.0 (0.4)

Figure S2. Optimized structures and relative Gibbs free energies of different conformers of neutral Chloroquine (CQ) and in the gas phase and in aqueous solution (the numbers in the parenthesis). Relative energies are in kJ mol^{-1}.

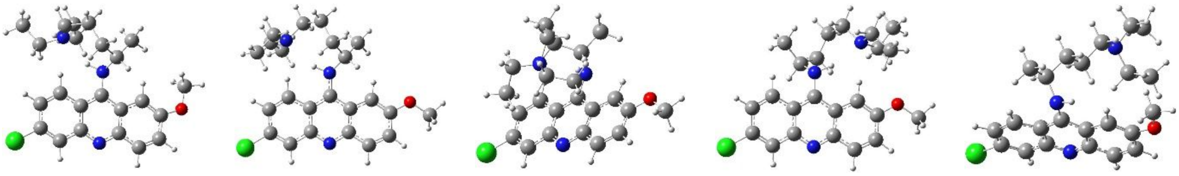

QR-a 7.1 (7.1) | **QR-b** 16.0 (17.6) | **QR-c** 1.3 (2.2) | **QR-d** 12.6 (15.6) | **QR-e** 0.0 (0.0)

Figure S3. Optimized structures and relative Gibbs free energies of different conformers of neutral Quinacrine (QR) and in the gas phase and in aqueous solution (the numbers in the parenthesis). Relative energies are in kJ mol^{-1}.

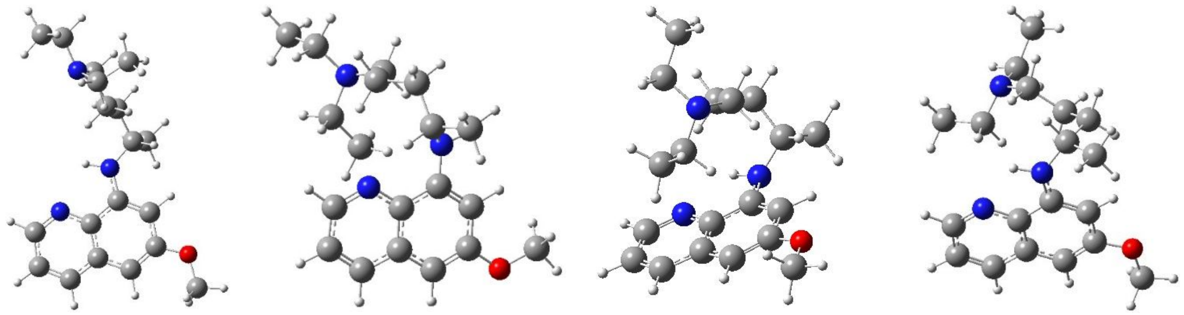

**PQ-a**	**PQ-b**	**PQ-c**	**PQ-d**
6.8	38.7	0.0	12.2
(3.5)	(40.6)	(0.0)	(13.2)

Figure S4. Optimized structures and relative Gibbs free energies of different conformers of neutral Pamaquine (PQ) and in the gas phase and in aqueous solution (the numbers in the parenthesis). Relative energies are in kJ mol^{-1}.

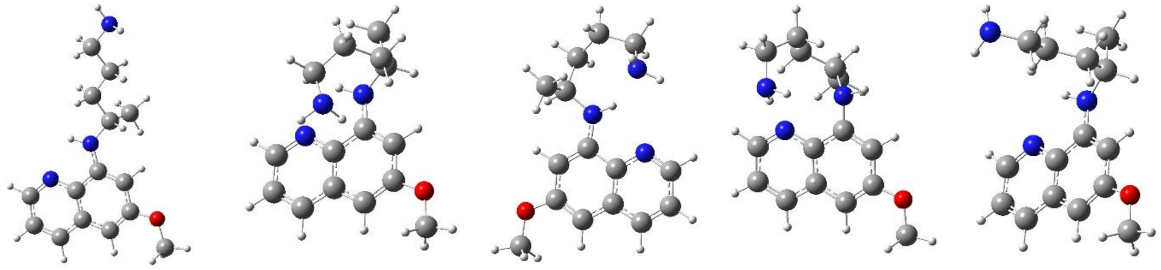

**PR-a**	**PR-b**	**PR-c**	**PR-d**	**PR-e**
1.2	0.0	2.9	43.7	0.9
(0.0)	(3.6)	(7.6)	(41.1)	(0.5)

Figure S5. Optimized structures and relative Gibbs free energies of different conformers of neutral Primaquine (PR) and in the gas phase and in aqueous solution (the numbers in the parenthesis). Relative energies are in kJ mol^{-1}.

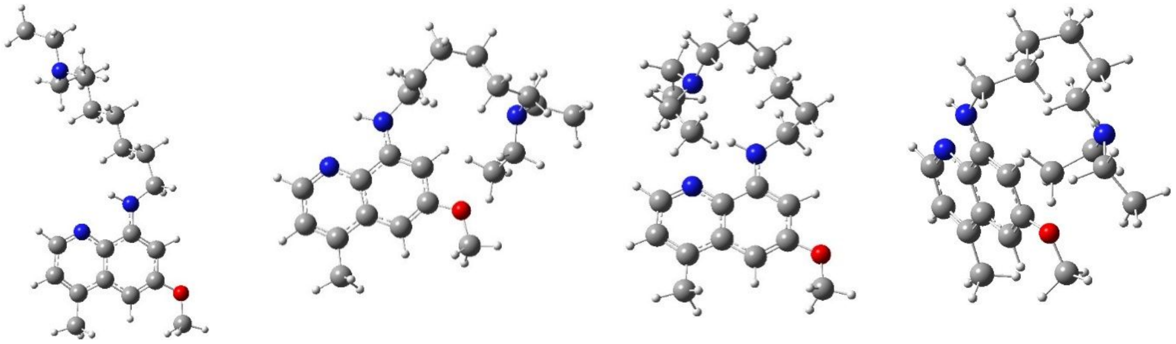

**SQ-a**	**SQ-b**	**SQ-c**	**SQ-d**
8.4	6.8	0.0	1.3
(11.7)	(12.6)	(2.3)	(0.0)

Figure S6. Optimized structures and relative Gibbs free energies of different conformers of neutral Sitamaquine (SQ) and in the gas phase and in aqueous solution (the numbers in the parenthesis). Relative energies are in kJ mol^{-1}.

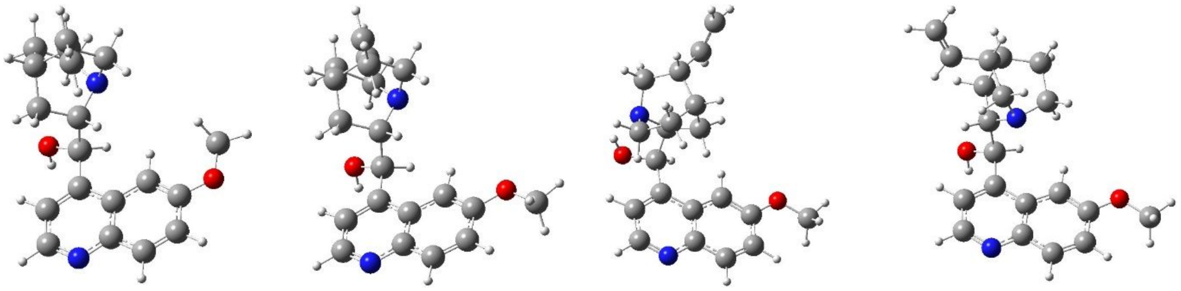

QN-a	QN-b	QN-c	QN-d
0.0	4.7	6.8	14.4
(0.0)	(3.4)	(6.8)	(7.4)

Figure S7. Optimized structures and relative Gibbs free energies of different conformers of neutral Quinine (QN) and in the gas phase and in aqueous solution (the numbers in the parenthesis). Relative energies are in kJ mol^{-1}.

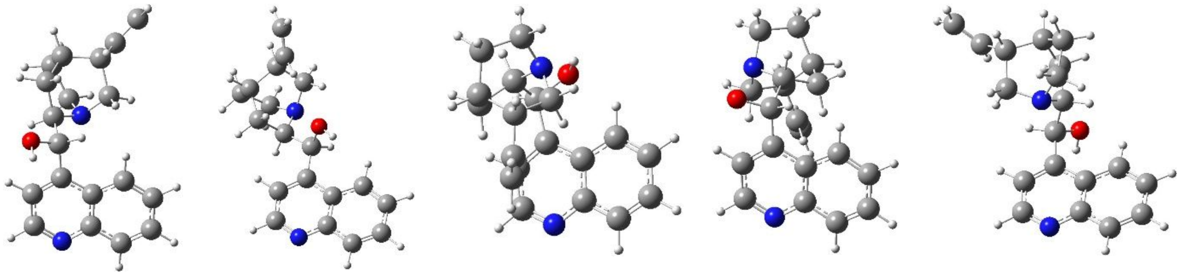

CIN-a	CIN-b	CIN-c	CIN-d	CIN-e
9.8	18.6	13.4	0.0	7.3
(3.2)	(19.1)	(9.0)	(0.0)	(3.8)

Figure S8. Optimized structures and relative Gibbs free energies of different conformers of neutral Cinchonine (CIN) and in the gas phase and in aqueous solution (the numbers in the parenthesis). Relative energies are in kJ mol^{-1}.

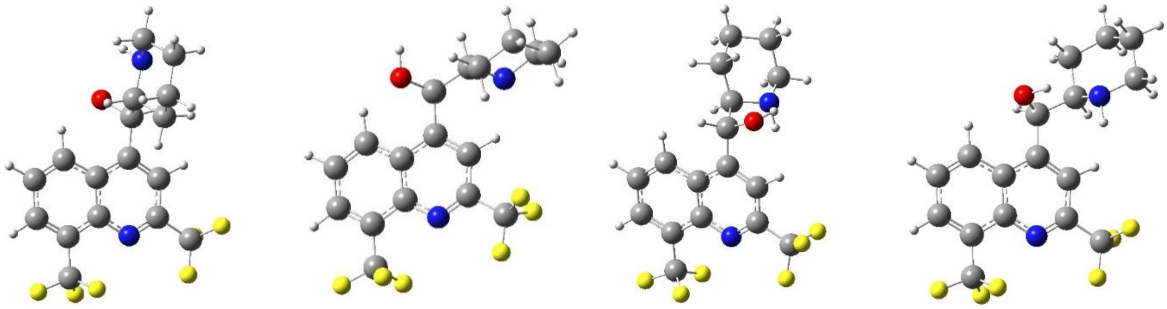

MQ-a	MQ-b	MQ-c	MQ-d
7.9	32.3	0.0	10.2
(8.5)	(26.8)	(0.0)	(14.5)

Figure S9. Optimized structures and relative Gibbs free energies of different conformers of neutral Mefloquine (MQ) and in the gas phase and in aqueous solution (the numbers in the parenthesis). Relative energies are in kJ mol^{-1}.

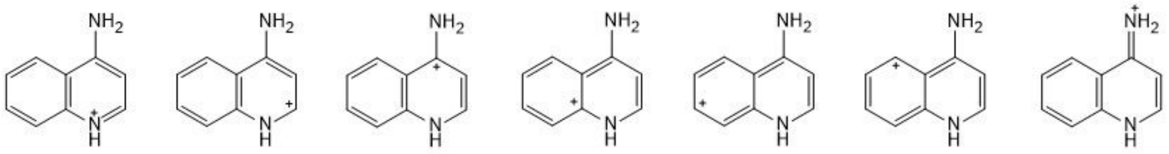

$[1,4+H]^+$-a $[1,4+H]^+$-b $[1,4+H]^+$-c $[1,4+H]^+$-d $[1,4+H]^+$-e $[1,4+H]^+$-f $[1,4+H]^+$-g

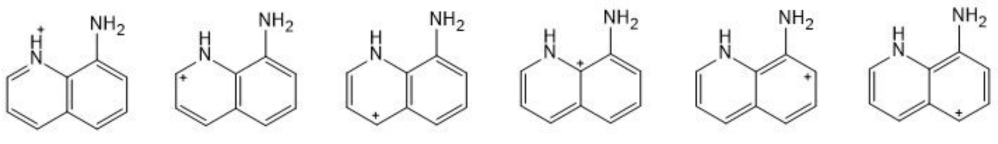

$[1,8+H]^+$-a $[1,8+H]^+$-b $[1,8+H]^+$-c $[1,8+H]^+$-d $[1,8+H]^+$-e $[1,8+H]^+$-f

Figure S10. The possible resonance structures for protonated forms of 1,4-aminoquinoline and 1,8-aminoquinoline, protonated at the ring nitrogen.

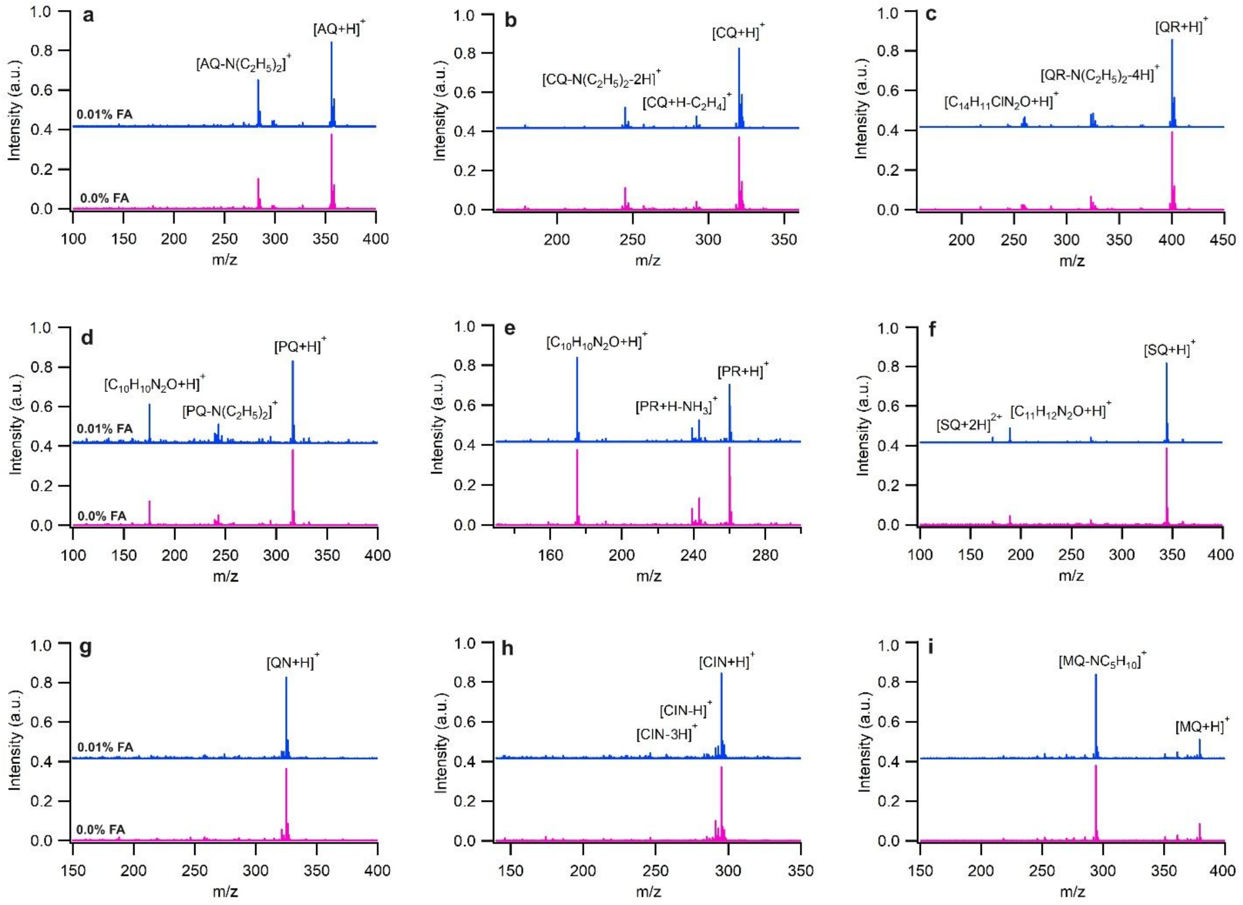

Figure S11. APCI mass spectra of (a) AQ, (b) CQ, (c) QR, (d) PQ, (e) PR, (f) SQ, (g) QN, (h) ClN, and (i) MQ in aqueous solvent without and with 0.01% FA.

Table S1. The measured *m/z* for the antimalarial drugs and their difference to theoretical masses (in ppm). Abbreviations: Amodiaquine (AQ), Cinchonine (CIN), Chloroquine (CQ), Mefloquine (MQ), Pamaquine (PQ), Primaquine (PR), Quinacrine (QR), Quinine (QN), and Sitamaquine (SQ).

**Ion**	**Formula**	**Measured *m/z***	**Difference (ppm)**
$[AQ+H]^+$	$[C_{20}H_{22}ClN_3O+H]^+$	356.1510	-3.98
$[AQ+2H]^{2+}$	$[C_{20}H_{22}ClN_3O+2H]^{2+}$	178.5788	-5.86
$[AQ-N(C_2H_5)_2]^+$	$[C_{16}H_{12}ClN_2O]^+$	283.0632	-0.24
$[CIN+H]^+$	$[C_{19}H_{22}N_2O+H]^+$	295.1817	4.10
$[CIN+2H]^{2+}$	$[C_{19}H_{22}N_2O+2H]^+$	148.0928	-7.31
$[CQ+H]^+$	$[C_{18}H_{26}ClN_3+H]^+$	320.1878	-3.30
$[CQ+2H]^{2+}$	$[C_{18}H_{26}ClN_3+2H]^{2+}$	160.5989	5.36
$[CQ-C_2H_3]^+$	$[C_{16}H_{23}ClN_3]^+$	292.1558	-5.83
$[CQ-N(C_2H_5)_2]^+$	$[C_{14}H_{16}ClN_2]^+$	247.1008	4.64
$[CQ-N(C_2H_5)_2-2H]^+$	$[C_{14}H_{14}ClN_2]^+$	245.0851	4.48
$[MQ+H]^+$	$[C_{17}H_{16}F_6N_2O+H]^+$	379.1225	-3.85
$[MQ+H-H_2O]^+$	$[C_{17}H_{15}F_6N_2]^+$	361.1130	-1.09
$[MQ-NC_5H_{10}]^+$	$[C_{12}H_6F_6NO]^+$	294.0341	-2.41
$[PQ+H]^+$	$[C_{19}H_{29}N_3O+H]^+$	316.2397	4.30
$[PQ+2H]^{2+}$	$[C_{19}H_{29}N_3O+2H]^{2+}$	158.6235	4.36
$[PQ-N(C_2H_5)_2]^+$	$[C_{15}H_{19}N_2O]^+$	243.1503	4.57
$[PR+H]^+$	$[C_{15}H_{21}N_3O+H]^+$	260.1755	-0.92
$[PR+H-NH_3]^+$	$[C_{15}H_{19}N_2O]^+$	243.1491	-0.37
$[QR+H]^+$	$[C_{23}H_{30}ClN_3O+H]^+$	400.2135	-3.79
$[QR+2H]^{2+}$	$[C_{23}H_{30}ClN_3O+2H]^{2+}$	200.6103	-4.22
$[QR-N(C_2H_5)_2-4H]^+$	$[C_{19}H_{16}ClN_2O]^+$	323.0928	-5.47
$[QN+H]^+$	$[C_{20}H_{24}N_2O_2+H]^+$	325.1895	-4.78
$[QN+2H]^{2+}$	$[C_{20}H_{24}N_2O_2+2H]^{2+}$	163.0981	-6.53
$[SQ+H]^+$	$[C_{21}H_{33}N_3O+H]^+$	344.2684	-3.6
$[SQ+2H]^{2+}$	$[C_{21}H_{33}N_3O+2H]^{2+}$	172.6393	4.88
$[C_{10}H_{10}N_2O+H]^+$	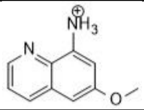	175.0857	-5.08
$[C_{11}H_{10}N_2O+H]^+$	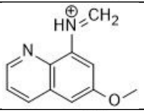	187.0862	-2.08
$[C_{14}H_{11}ClN_2O+H]^+$	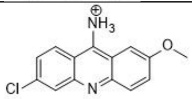	259.0619	-5.26
$[C_{11}H_{12}N_2O+H]^+$	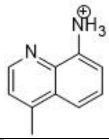	189.1013	-4.97
$[C_{10}H_9NO]^+$	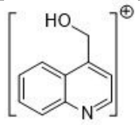	159.0675	-2.30

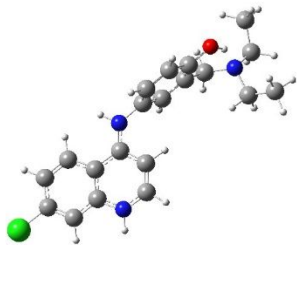

[AQ+H]+-a1

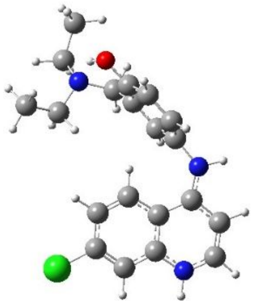

[AQ+H]+-a2

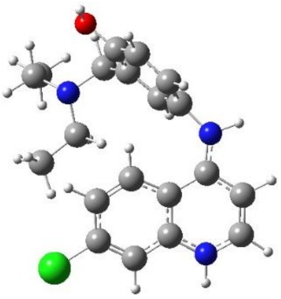

[AQ+H]+-a3

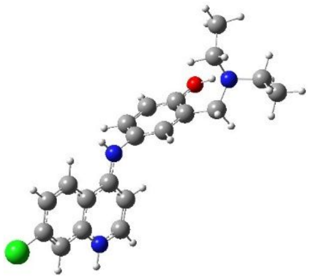

[AQ+H]+-a4

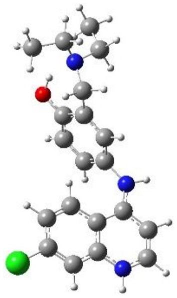

[AQ+H]+-a5

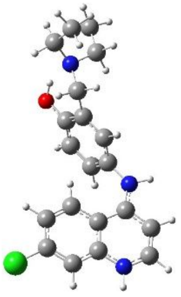

[AQ+H]+-a6

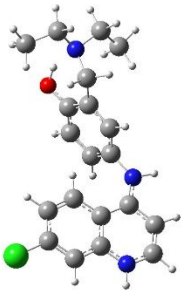

[AQ+H]+-a7

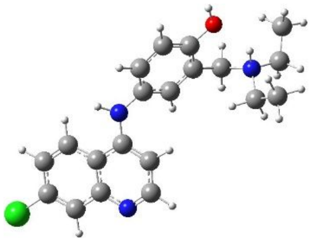

[AQ+H]+-b1

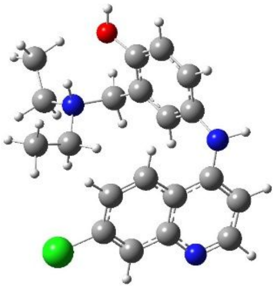

[AQ+H]+-b2

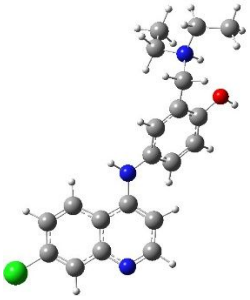

[AQ+H]+-b3

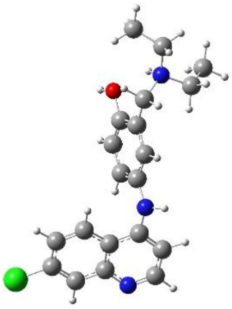

[AQ+H]+-b4

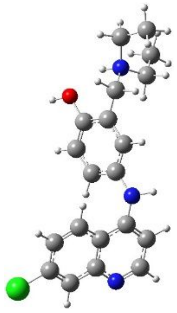

[AQ+H]+-b5

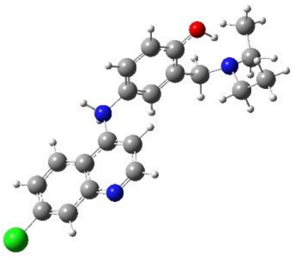

[AQ+H]+-c1

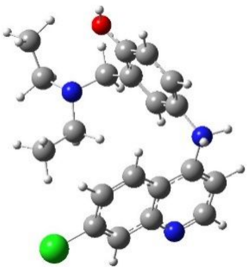

[AQ+H]+-c2

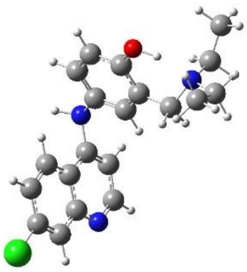

[AQ+H]+-c3

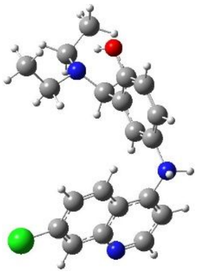

[AQ+H]+-c4

Figure S12. Optimized structures of protomers and conformers of mono-protonated Amodiaquine, [AQ+H]+, in the gas phase. Letters **a**, **b**, and **c** indicate different sites of protonation.

Table S2. The calculated relative Gibbs free energies of different protomers and conformers of $[AQ+H]^+$ in the gas phase and in aqueous solution, their relative abundances in solution, and their calculated CCS_{N2} values. (Experimental $^{DT}CCS_{N2}$ = 193.7 and 186.1 Å^2 and computed Boltzmann-weighted CCS_{N2}=192.7 Å^2).

Ion	ΔG in gas phase (kJ mol^{-1})	ΔG in water (kJ mol^{-1})	Rel. Abundance	CCS_{N2} (Å^2)
$[AQ+H]^+$-a1	0.0	0.0	0.869242	192.9
$[AQ+H]^+$-a2	13.7	10.7	0.0116	187.2
$[AQ+H]^+$-a3	50.1	36.0	4.28×10^{-7}	180.8
$[AQ+H]^+$-a4	1.3	6.9	0.053732	193.6
$[AQ+H]^+$-a5	15.3	6.6	0.060645	190.4
$[AQ+H]^+$-a6	15.7	14.9	0.002131	190.9
$[AQ+H]^+$-a7	21.7	17.4	0.000777	189.2
$[AQ+H]^+$-b1	52.8	19.2	0.000376	196.8
$[AQ+H]^+$-b2	42.7	17.0	0.000913	181.6
$[AQ+H]^+$-b4	53.4	18.5	0.000499	196.8
$[AQ+H]^+$-b5	49.5	23.2	7.49×10^{-5}	196.5
$[AQ+H]^+$-b6	52.1	28.6	8.48×10^{-6}	195.7
$[AQ+H]^+$-c1	136.5	110.2	4.28×10^{-20}	194.7
$[AQ+H]^+$-c2	178.9	136.9	8.99×10^{-25}	184.5
$[AQ+H]^+$-c3	136.5	109.8	5.03×10^{-20}	194.1
$[AQ+H]^+$-c4	136.9	106.4	1.98×10^{-19}	188.8

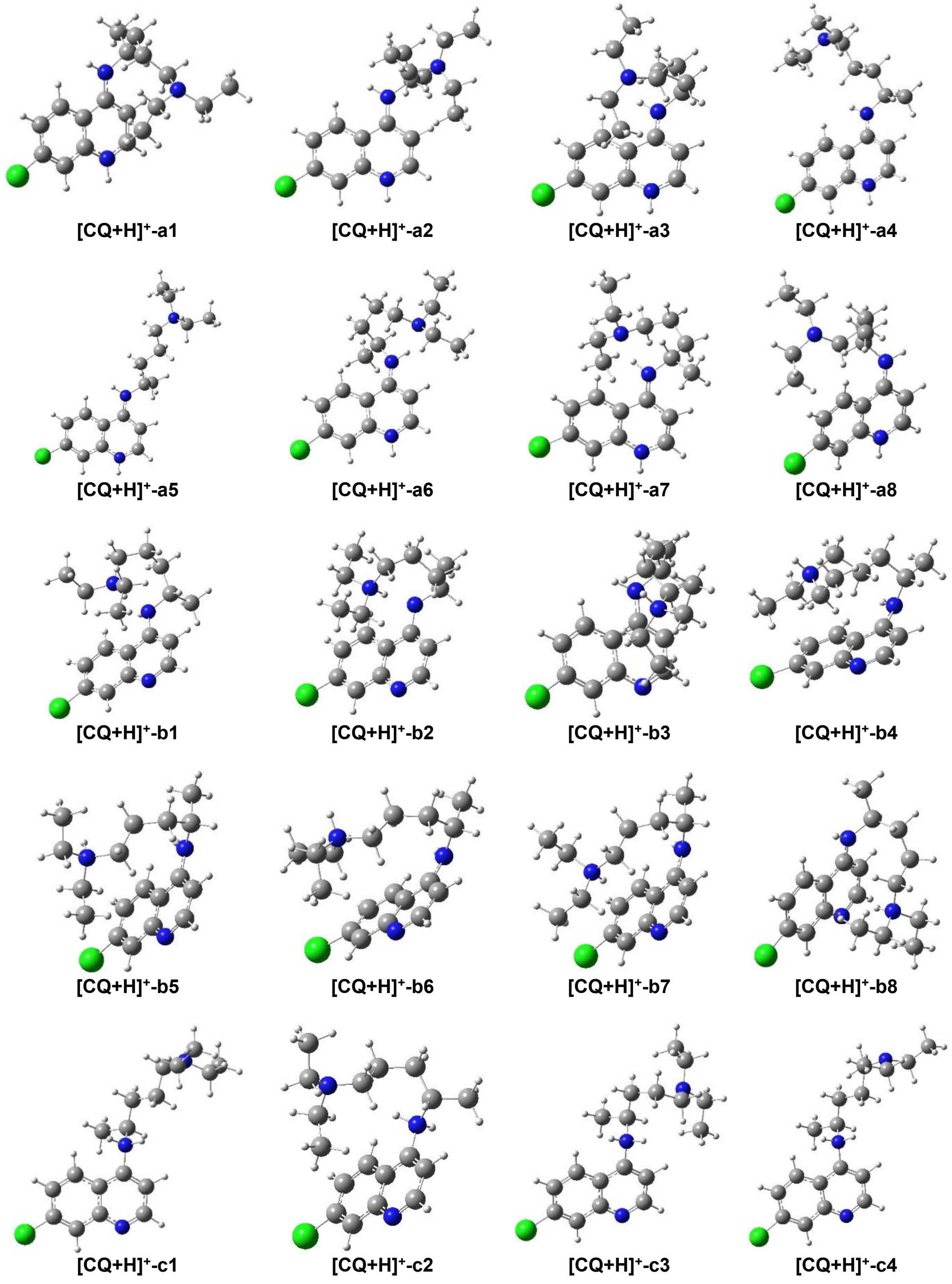

Figure S13. Optimized structures of protomers and conformers of mono-protonated Chloroquine, [CQ+H]$^+$, in the gas phase. Letters **a**, **b**, and **c** indicate different sites of protonation.

Table S3. The calculated relative Gibbs free energies of different protomers and conformers of $[CQ+H]^+$ in the gas phase and in aqueous solution, their relative abundances in solution, and their calculated CCS_{N2} values. (Experimental $^{DT}CCS_{N2}$ = 175.3 Å^2 and computed Boltzmann-weighted CCS_{N2}=173.1 Å^2).

Ion	ΔG in gas phase (kJ mol^{-1})	ΔG in water (kJ mol^{-1})	Rel. Abundance	CCS_{N2} (Å^2)
$[CQ+H]^+$-a1	8.9	12.7	0.005695	179.7
$[CQ+H]^+$-a2	0.0	12.0	0.007554	179.6
$[CQ+H]^+$-a3	17.9	20.9	0.000208	178.1
$[CQ+H]^+$-a4	14.7	15.69	0.001705	185.3
$[CQ+H]^+$-a5	14.4	18.19	0.000622	192.3
$[CQ+H]^+$-a6	25.4	51.09	1.07×10^{-9}	173.8
$[CQ+H]^+$-a7	1.6	21.6	0.000157	169.2
$[CQ+H]^+$-a8	17.1	30.5	4.33×10^{-6}	173.0
$[CQ+H]^+$-b1	6.4	0.0	0.956291	173.0
$[CQ+H]^+$-b2	10.3	15.5	0.001841	170.1
$[CQ+H]^+$-b3	30.6	10.8	0.012258	174.8
$[CQ+H]^+$-b4	38.2	16.6	0.001181	171.6
$[CQ+H]^+$-b5	36.5	17.2	0.000927	172.0
$[CQ+H]^+$-b6	31.8	16.7	0.001134	169.7
$[CQ+H]^+$-b7	24.4	16.6	0.001181	166.8
$[CQ+H]^+$-b8	15.4	11.5	0.009242	169.5
$[CQ+H]^+$-c1	131.7	113.4	1.3×10^{-20}	191.0
$[CQ+H]^+$-c2	140.7	117.2	2.8×10^{-21}	175.7
$[CQ+H]^+$-c3	120.1	111.9	2.37×10^{-20}	179.6
$[CQ+H]^+$-c4	129.4	112.1	2.19×10^{-20}	190.1

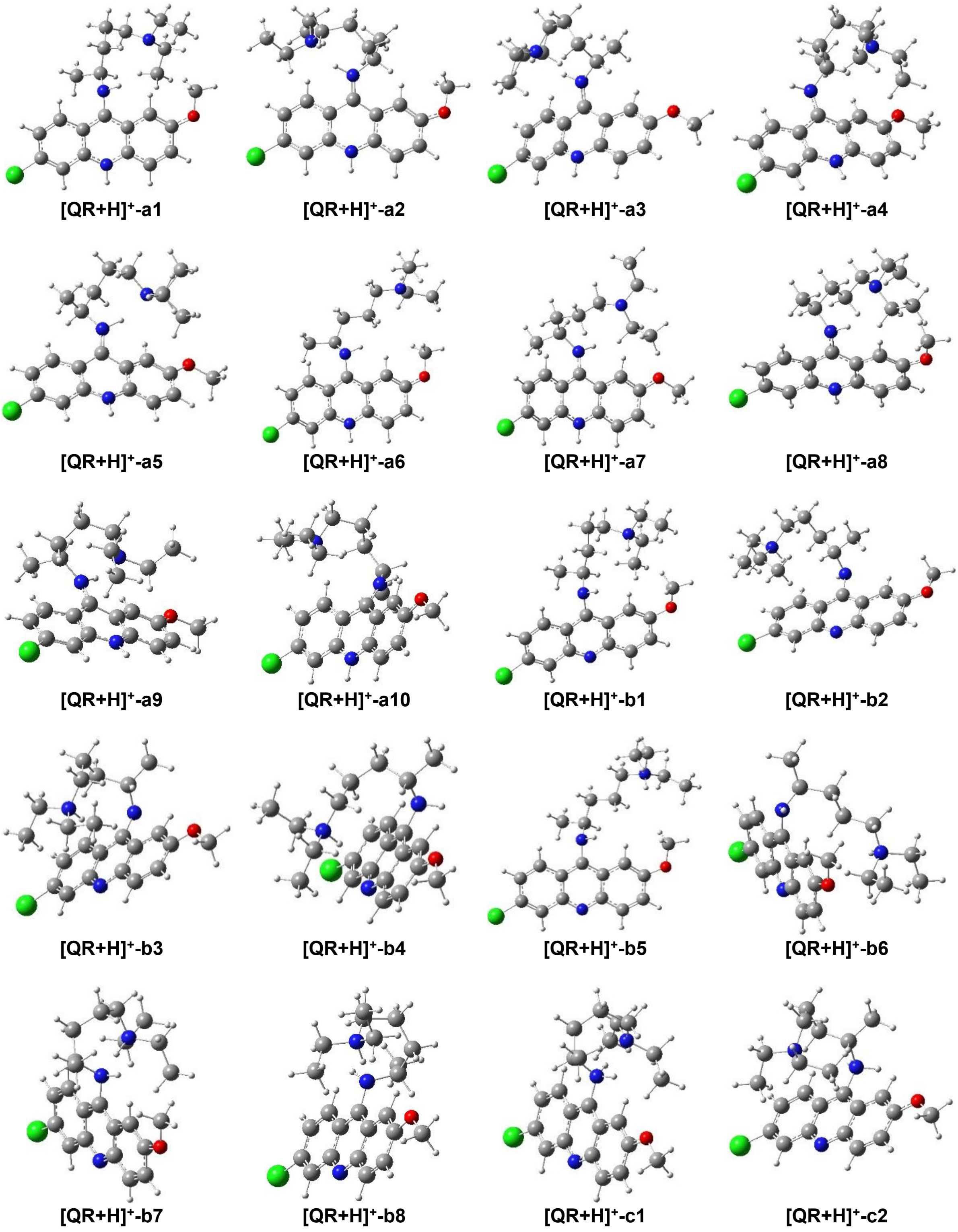

Figure S14. Optimized structures of protomers and conformers of mono-protonated Quinacrine, [QR+H]$^{+}$, in the gas phase. Letters **a**, **b**, and **c** indicate different sites of protonation.

Table S4. The calculated relative Gibbs free energies of different protomers and conformers of $[QR+H]^+$ in the gas phase and in aqueous solution, their relative abundances in solution, and their calculated CCS_{N2} values. (Experimental ${}^{DT}CCS_{N2}$ = 195.3 Å^2 and computed Boltzmann-weighted CCS_{N2}=192.0 Å^2)

Ion	ΔG in gas phase (kJ mol^{-1})	ΔG in water (kJ mol^{-1})	Rel. Abundance	CCS_{N2} (Å^2)
$[QR+H]^+$-a1	52.7	46.3	6.23963×10^{-9}	191.7
$[QR+H]^+$-a2	0.0	0.0	0.807237367	191.4
$[QR+H]^+$-a3	4.7	8.5	0.026168941	192.4
$[QR+H]^+$-a4	15.7	11.5	0.007801549	193.8
$[QR+H]^+$-a5	9.4	11.7	0.007196817	190.7
$[QR+H]^+$-a6	66.1	52.3	5.5456×10^{-10}	194.7
$[QR+H]^+$-a7	49.4	40.9	5.51123×10^{-8}	202.3
$[QR+H]^+$-a8	26.7	13.0	0.004259692	195.3
$[QR+H]^+$-a9	30.8	28.3	8.88773×10^{-6}	190.2
$[QR+H]^+$-a10	39.4	38.3	1.57316×10^{-7}	196.5
$[QR+H]^+$-b1	90.6	35.8	4.31298×10^{-7}	200.5
$[QR+H]^+$-b2	71.1	36.2	3.67026×10^{-7}	192.9
$[QR+H]^+$-b3	44.4	19.9	0.000263321	191.1
$[QR+H]^+$-b4	38.8	30.2	4.1296×10^{-6}	189.8
$[QR+H]^+$-b5	103.7	27.3	1.33043×10^{-5}	209.1
$[QR+H]^+$-b6	54.8	5.8	0.077773376	195.0
$[QR+H]^+$-b7	37.8	6.1	0.068908123	195.3
$[QR+H]^+$-b8	46.5	19.1	0.00036362	196.8
$[QR+H]^+$-c2	79.1	66.1	2.11916×10^{-12}	196.9
$[QR+H]^+$-c3	111.6	82.9	2.4142×10^{-15}	192.1

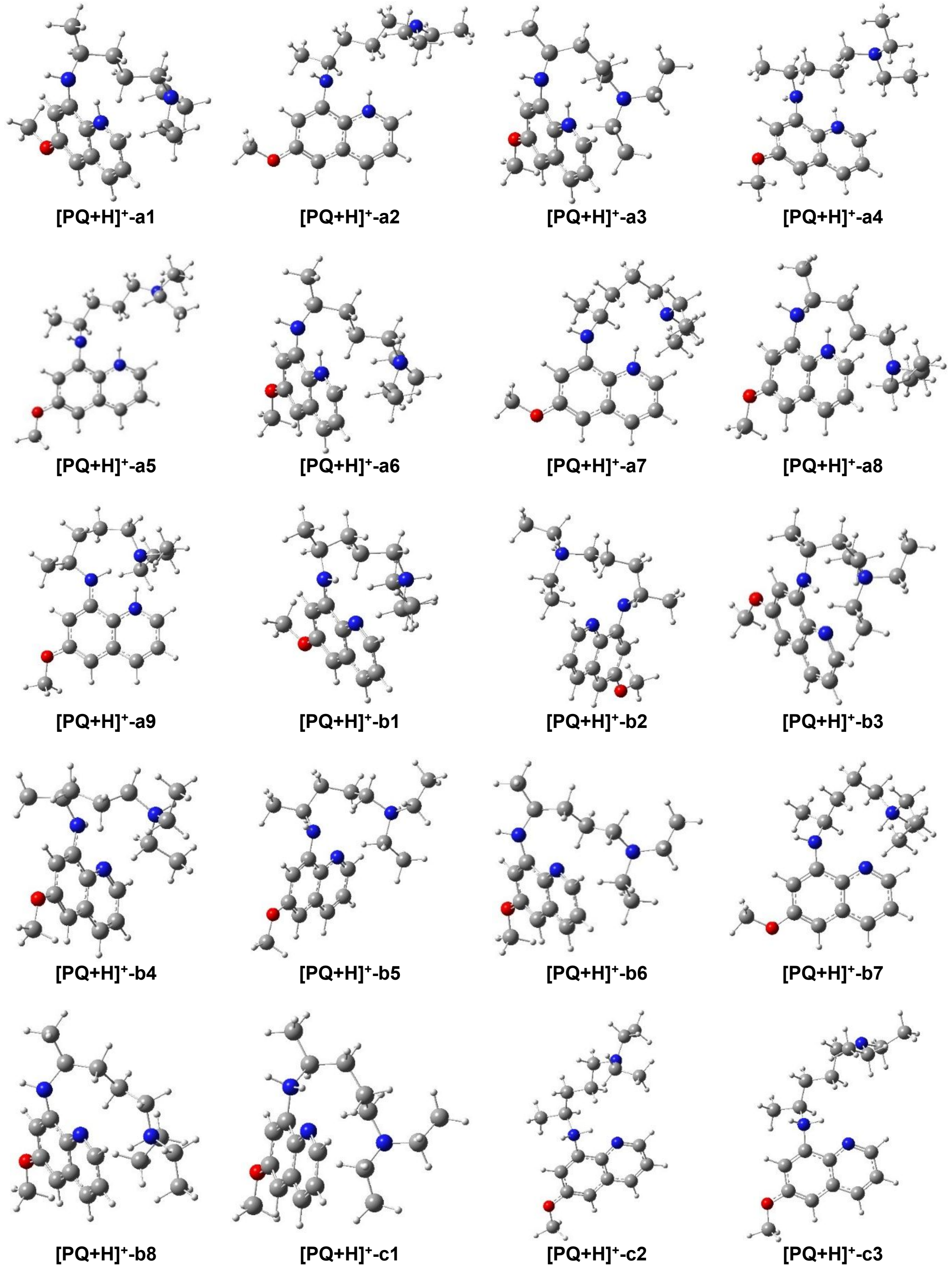

Figure S15. Optimized structures of protomers and conformers of mono-protonated Pamaquine, $[PQ+H]^+$, in the gas phase. Letters **a**, **b**, and **c** indicate different sites of protonation.

Table S5. The calculated relative Gibbs free energies of different protomers and conformers of $[PQ+H]^+$ in the gas phase and in aqueous solution, their relative abundances in solution, and their calculated CCS_{N2} values. (Experimental $^{DT}CCS_{N2}$ = 175.6 Å^2 and computed Boltzmann-weighted CCS_{N2}=175.5 Å^2)

Ion	ΔG in gas phase (kJ mol^{-1})	ΔG in water (kJ mol^{-1})	Rel. Abundance	CCS_{N2} (Å^2)
$[PQ+H]^+$-a1	52.4	63.6	7.11879×10^{-12}	176.4
$[PQ+H]^+$-a2	50.9	61.8	1.47153×10^{-11}	183.3
$[PQ+H]^+$-a3	45.2	49.6	2.01944×10^{-9}	176.3
$[PQ+H]^+$-a4	57.3	65.1	3.8869×10^{-12}	177.2
$[PQ+H]^+$-a5	37.6	56.6	1.199×10^{-10}	181.8
$[PQ+H]^+$-a6	44.6	53.4	4.35977×10^{-10}	174.4
$[PQ+H]^+$-a7	23.3	56.1	1.46696×10^{-10}	171.5
[PQ+H]-a8	46.1	68.3	1.06895×10^{-12}	171.5
$[PQ+H]^+$-a9	9.9	34.4	9.29604×10^{-7}	171.2
$[PQ+H]^+$-b1	20.9	12.0	0.007812997	175.0
$[PQ+H]^+$-b2	24.5	28.9	8.54885×10^{-6}	180.6
$[PQ+H]^+$-b3	0.0	0.0	0.989097182	175.5
$[PQ+H]^+$-b4	12.9	14.4	0.002967108	177.4
$[PQ+H]^+$-b5	12.4	23.5	7.55087×10^{-5}	181.6
$[PQ+H]^+$-b6	50.2	41.5	5.30109×10^{-8}	179.2
$[PQ+H]^+$-b7	13.0	25.2	3.80324×10^{-5}	172.9
$[PQ+H]^+$-b8	48.3	50.3	1.52261×10^{-9}	171.0
$[PQ+H]^+$-c1	50.2	58.8	4.93599×10^{-11}	176.5
$[PQ+H]^+$-c2	55.0	67.6	1.41775×10^{-12}	184.1
$[PQ+H]^+$-c3	56.9	64.4	5.15519×10^{-12}	184.3

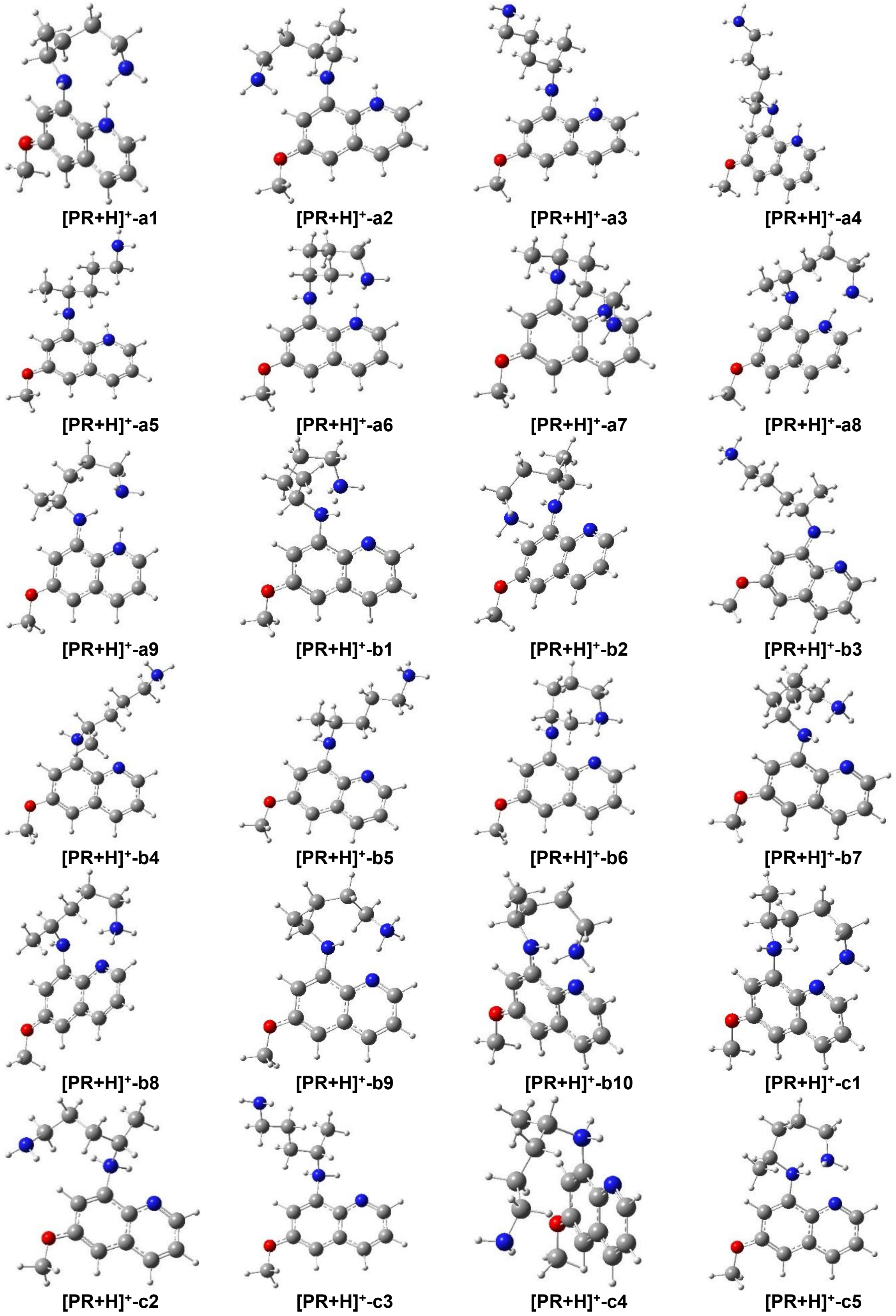

Figure S16. Optimized structures of protomers and conformers of mono-protonated Primaquine, $[PR+H]^+$, in the gas phase. Letters **a**, **b**, and **c** indicate different sites of protonation.

Table S6. The calculated relative Gibbs free energies of different protomers and conformers of $[PR+H]^+$ in the gas phase and in aqueous solution, their relative abundances in solution, and their calculated CCS_{N2} values. (Experimental $^{DT}CCS_{N2}$ = 161.7 Å^2 and computed Boltzmann-weighted CCS_{N2}=160.4 Å^2).

Ion	ΔG in gas phase (kJ mol^{-1})	ΔG in water (kJ mol^{-1})	Rel. Abundance	CCS_{N2} (Å^2)
$[PR+H]^+$-a1	49.8	51.9	7.25986×10^{-10}	157.9
$[PR+H]^+$-a2	54.5	59.9	2.87948×10^{-11}	158.8
$[PR+H]^+$-a3	40.9	44.1	1.6885×10^{-8}	166.4
$[PR+H]^+$-a4	39.0	46.6	6.15882×10^{-9}	165.1
$[PR+H]^+$-a5	46.2	53.2	4.29701×10^{-10}	161.3
$[PR+H]^+$-a6	14.4	39.0	1.32139×10^{-7}	152.3
$[PR+H]^+$-a7	52.3	56.4	1.18174×10^{-10}	155.5
$[PR+H]^+$-a8	7.2	32.7	1.67804×10^{-6}	152.7
$[PR+H]^+$-a9	3.0	23.8	6.08272×10^{-5}	155.1
$[PR+H]^+$-b1	11.9	6.3	0.070815646	161.1
$[PR+H]^+$-b2	58.6	34.5	8.11781×10^{-7}	158.4
$[PR+H]^+$-b3	83.3	11.2	0.009809356	182.3
$[PR+H]^+$-b4	115.7	51.7	7.86989×10^{-7}	179.1
$[PR+H]^+$-b5	123.1	55.7	1.56733×10^{-10}	178.4
$[PR+H]^+$-b6	24.5	26.6	1.96577×10^{-5}	156.5
$[PR+H]^+$-b7	0.0	0.0	0.899291346	160.1
$[PR+H]^+$-b8	6.6	15.9	0.001472964	155.5
$[PR+H]^+$-b9	25.9	18.3	0.000559381	158.3
$[PR+H]^+$-b10	25.7	9.7	0.01796566	159.7
$[PR+H]^+$-c1	56.9	65.0	3.67947×10^{-12}	156.7
$[PR+H]^+$-c2	58.5	74.9	6.7809×10^{-14}	156.1
$[PR+H]^+$-c3	47.6	49.4	1.99036×10^{-9}	162.8
$[PR+H]^+$-c4	60.2	54.7	2.34619×10^{-10}	159.7
$[PR+H]^+$-c5	9.1	31.7	2.51191×10^{-6}	154.0

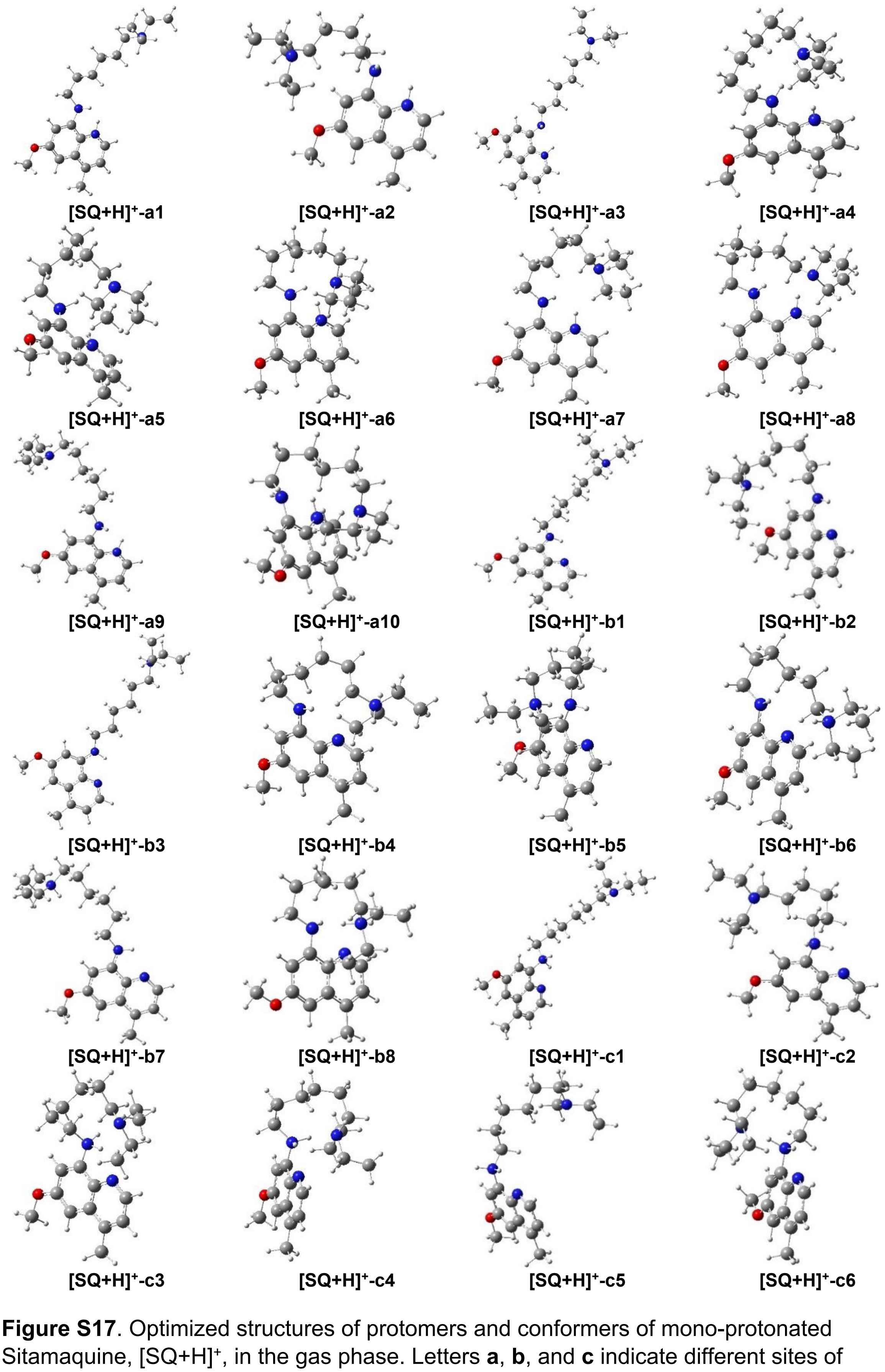

Figure S17. Optimized structures of protomers and conformers of mono-protonated Sitamaquine, $[SQ+H]^+$, in the gas phase. Letters **a**, **b**, and **c** indicate different sites of protonation.

Table S7. The calculated relative Gibbs free energies of different protomers and conformers of $[SQ+H]^+$ in the gas phase and in aqueous solution, their relative abundances in solution, and their calculated CCS_{N2} values. (Experimental ${}^{DT}CCS_{N2}$ = 182.9 Å^2 and computed Boltzmann-weighted CCS_{N2}=180.0 Å^2).

Ion	ΔG in gas phase (kJ mol^{-1})	ΔG in water (kJ mol^{-1})	Rel. Abundance	CCS_{N2} (Å^2)
$[SQ+H]^+$-a1	78.3	57.2	5.43283×10^{-11}	206.4
$[SQ+H]^+$-a2	74.1	61.5	9.58646×10^{-12}	188.5
$[SQ+H]^+$-a3	82.7	59.7	1.98162×10^{-11}	207.2
$[SQ+H]^+$-a4	42.0	47.2	3.06933×10^{-9}	178.1
$[SQ+H]^+$-a5	70.7	51.9	4.60888×10^{-10}	178.8
$[SQ+H]^+$-a6	45.3	46.2	4.59458×10^{-9}	180.3
$[SQ+H]^+$-a7	65.8	57.5	4.81355×10^{-11}	184.8
$[SQ+H]^+$-a8	42.7	48.9	1.54597×10^{-9}	182.3
$[SQ+H]^+$-a9	73.7	59.6	2.0632×10^{-11}	201.9
$[SQ+H]^+$-a10	66.2	54.7	1.48947×10^{-10}	177.1
$[SQ+H]^+$-b1	68.1	13.1	0.002893505	211.2
$[SQ+H]^+$-b2	45.1	13.9	0.00209538	186.1
$[SQ+H]^+$-b3	71.3	15.7	0.001013677	215.5
$[SQ+H]^+$-b4	21.4	8.5	0.018507699	180.1
$[SQ+H]^+$-b5	0.0	0.0	0.570909859	179.8
$[SQ+H]^+$-b6	8.7	2.8	0.184502633	180.5
$[SQ+H]^+$-b7	65.3	12.8	0.003265764	206.0
$[SQ+H]^+$-b8	4.3	2.4	0.216811981	179.1
$[SQ+H]^+$-c1	92.0	71.7	1.56531×10^{-13}	205.8
$[SQ+H]^+$-c2	85.2	66.2	1.43949×10^{-12}	188.1
$[SQ+H]^+$-c3	32.31	51.5	5.41597×10^{-10}	177.8
$[SQ+H]^+$-c4	26.9	40.3	4.96519×10^{-8}	181.0
$[SQ+H]^+$-c5	83.7	73.3	8.20875×10^{-14}	196.3
$[SQ+H]^+$-c6	44.3	53.4	2.51648×10^{-10}	179.4

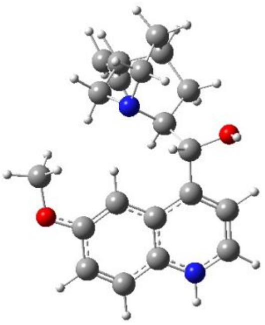
[QN+H]$^+$-a1
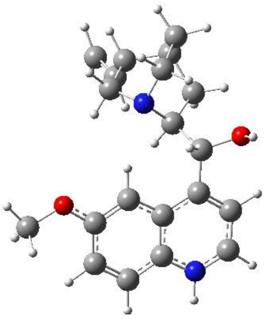
[QN+H]$^+$-a2
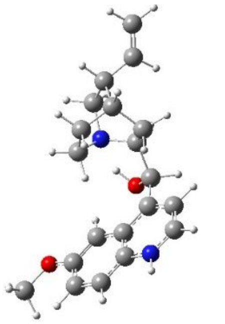
[QN+H]$^+$-a3
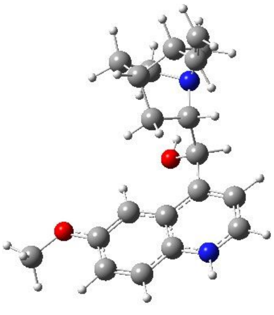
[QN+H]$^+$-a4
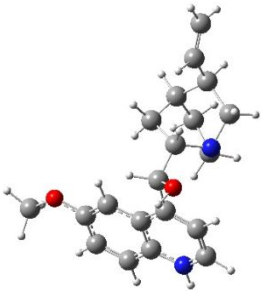
[QN+H]$^+$-a5
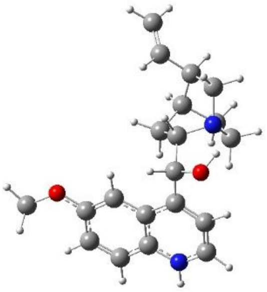
[QN+H]$^+$-a6
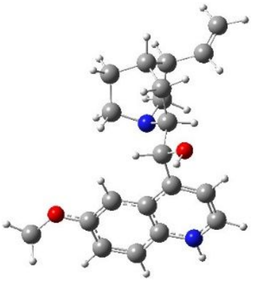
[QN+H]$^+$-a7
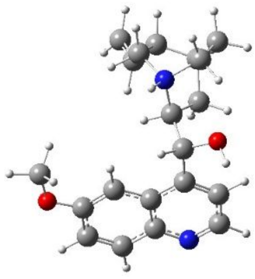
[QN+H]$^+$-b1
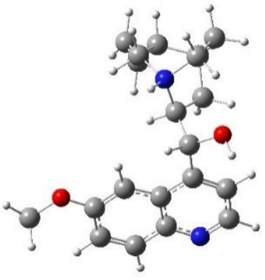
[QN+H]$^+$-b2
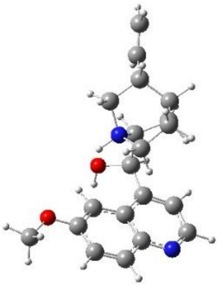
[QN+H]$^+$-b3
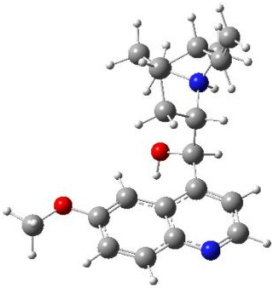
[QN+H]$^+$-b4
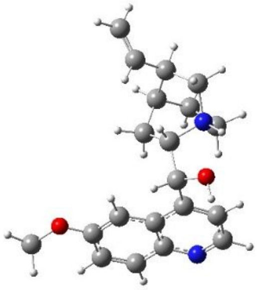
[QN+H]$^+$-b5
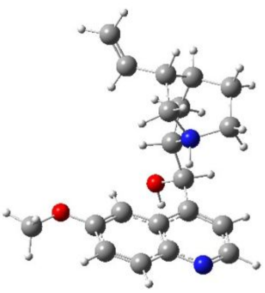
[QN+H]$^+$-b6
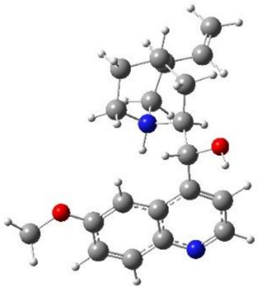
[QN+H]$^+$-b7
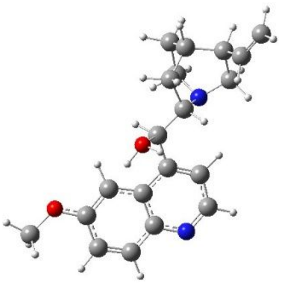
[QN+H]$^+$-c1
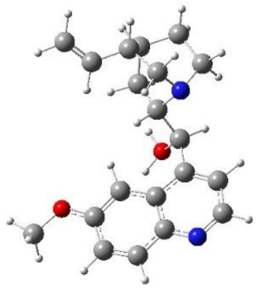
[QN+H]$^+$-c2

Figure S18. Optimized structures of protomers and conformers of mono-protonated Quinine, [QN+H]$^+$, in the gas phase. Letters **a**, **b**, and **c** indicate different sites of protonation (**c** indicates protonation at OH group).

Table S8. The calculated relative Gibbs free energies of different protomers and conformers of $[QN+H]^+$ in the gas phase and in aqueous solution, their relative abundances in solution, and their calculated CCS_{N2} values. (Experimental $^{DT}CCS_{N2}$ = 176.9 and 183.9 Å^2 and computed Boltzmann-weighted CCS_{N2}=174.9 Å^2).

Ion	ΔG in gas phase (kJ mol^{-1})	ΔG in water (kJ mol^{-1})	Rel. Abundance	CCS_{N2} (Å^2)
$[QN+H]^+$-a1	9.8	27.3	1.33095×10^{-5}	176.0
$[QN+H]^+$-a2	17.2	28.7	7.56622×10^{-6}	179.3
$[QN+H]^+$-a3	28.3	39.9	8.25314×10^{-8}	175.4
$[QN+H]^+$-a4	34.2	48.3	2.78563×10^{-9}	179.5
$[QN+H]^+$-a5	33.7	44.3	1.39872×10^{-8}	177.3
$[QN+H]^+$-a6	15.6	30.7	3.37657×10^{-6}	176.4
$[QN+H]^+$-a7	13.9	30.6	3.51557×10^{-6}	178.9
$[QN+H]^+$-b1	7.6	0.0	0.807551033	174.5
$[QN+H]^+$-b2	1.8	4.4	0.136861772	176.9
$[QN+H]^+$-b3	5.5	15.4	0.001618313	174.3
$[QN+H]^+$-b4	18.6	11.3	0.008460381	180.5
$[QN+H]^+$-b5	0.0	10.0	0.014293948	174.8
$[QN+H]^+$-b6	2.6	8.5	0.02617911	173.8
$[QN+H]^+$-b7	1.9	12.6	0.005007576	177.9
$[QN+H]^+$-c1	185.2	177.4	6.70463×10^{-32}	180.1
$[QN+H]^+$-c2	186.9	183.7	5.27963×10^{-33}	178.1

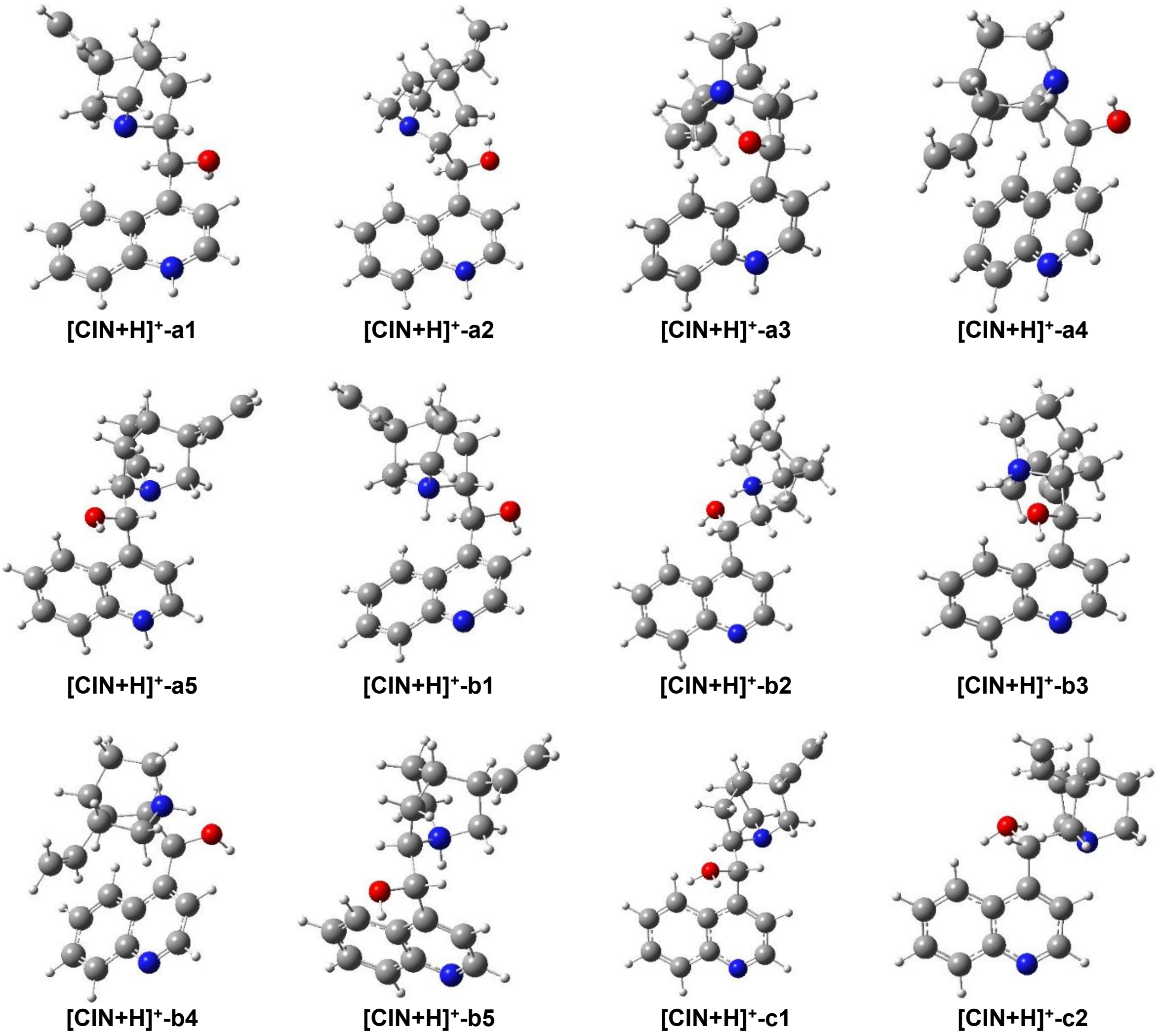

Figure S19. Optimized structures of protomers and conformers of mono-protonated Cinchonine, [CQ+H]$^+$, in the gas phase. Letters **a**, **b**, and **c** indicate different sites of protonation (**c** indicates protonation at OH group).

Table S9. The calculated relative Gibbs free energies of different protomers and conformers of $[CIN+H]^+$ in the gas phase and in aqueous solution, their relative abundances in solution, and their calculated CCS_{N2} values. (Experimental $^{DT}CCS_{N2}$ = 166.8, 169.8 and 176.2 Å^2 and computed Boltzmann-weighted CCS_{N2}=166.5 Å^2).

Ion	ΔG in gas phase (kJ mol^{-1})	ΔG in water (kJ mol^{-1})	Rel. Abundance	CCS_{N2} (Å^2)
$[CIN+H]^+$-a1	18.9	23.0	4.90373×10^{-5}	167.7
$[CIN+H]^+$-a2	21.5	22.5	5.99967×10^{-5}	168.9
$[CIN+H]^+$-a3	26.4	30.6	2.28556×10^{-6}	164.6
$[CIN+H]^+$-a4	14.9	19.7	0.000185649	168.0
$[CIN+H]^+$-a5	16.7	24.3	2.90245×10^{-5}	168.8
$[CIN+H]^+$-b1	5.8	3.0	0.15651716	167.5
$[CIN+H]^+$-b2	27.4	12.7	0.003126833	167.1
$[CIN+H]^+$-b3	5.0	5.8	0.050582115	164.3
$[CIN+H]^+$-b4	0.0	0.0	0.525009653	165.5
$[CIN+H]^+$-b5	6.9	1.7	0.264438246	168.2
$[CIN+H]^+$-c1	201.1	171.4	4.90436×10^{-31}	174.5
$[CIN+H]^+$-c2	195.4	164.4	8.26029×10^{-30}	171.6

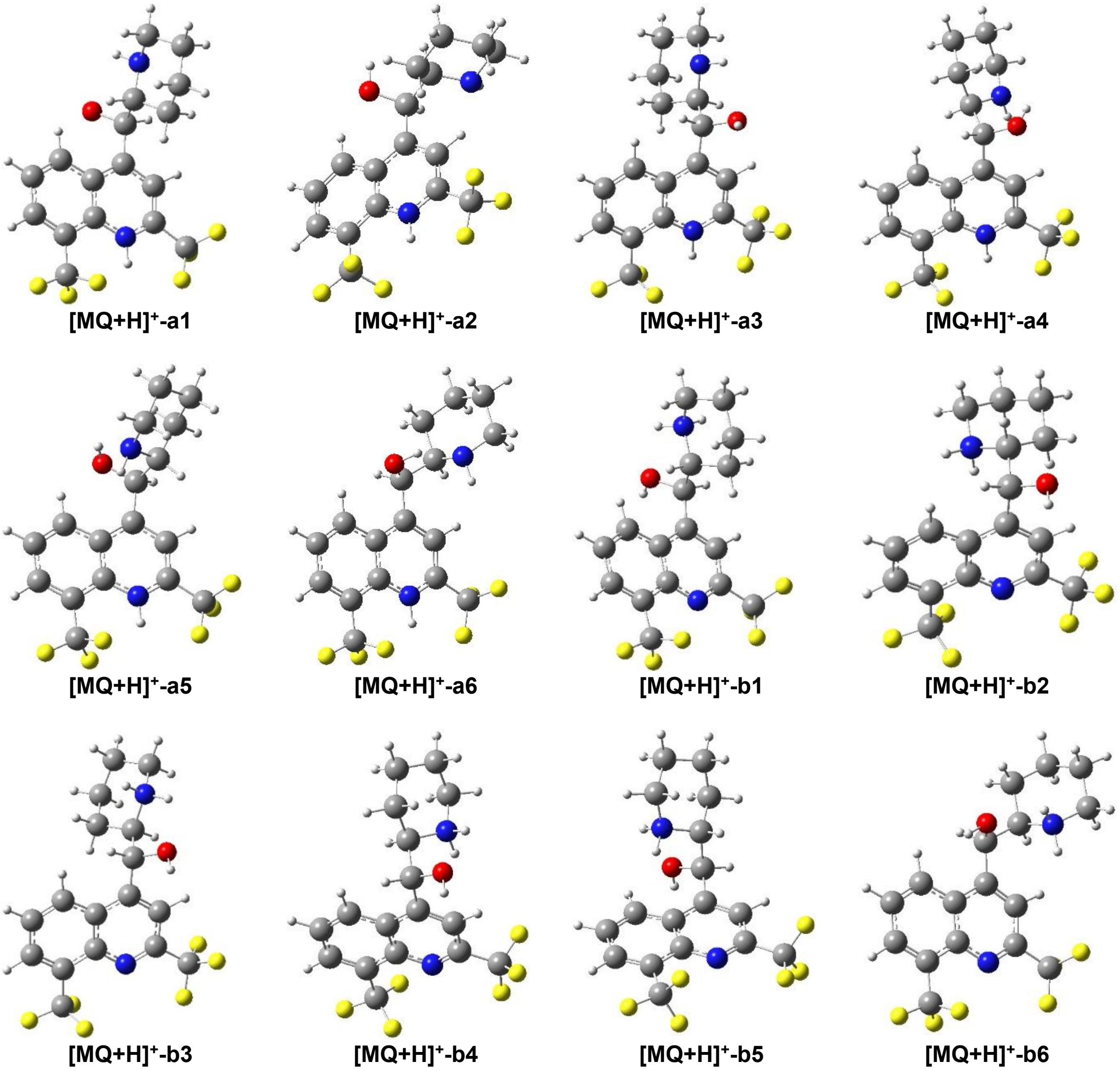

Figure S20. Optimized structures of protomers and conformers of mono-protonated mefloquine, $[MQ+H]^+$, in the gas phase. Letters **a** and **b** indicate different sites of protonation.

Table S10. The calculated relative Gibbs free energies of different protomers and conformers of $[MQ+H]^+$ in the gas phase and in aqueous solution, their relative abundances in solution, and their calculated CCS_{N2} values. (Experimental $^{DT}CCS_{N2}$ = 183.4 Å^2 and computed Boltzmann-weighted CCS_{N2}=179.2 Å^2).

Ion	ΔG in gas phase (kJ mol^{-1})	ΔG in water (kJ mol^{-1})	Rel. Abundance	CCS_{N2} (Å^2)
$[MQ+H]^+$-a1	38.3	82.7	1.54939×10^{-15}	170.5
$[MQ+H]^+$-a2	43.6	102.5	5.26216×10^{-19}	165.0
$[MQ+H]^+$-a3	38.8	83.9	9.54811×10^{-16}	170.3
$[MQ+H]^+$-a4	28.9	77.1	1.48351×10^{-14}	169.0
$[MQ+H]^+$-a5	43.8	89.5	9.97212×10^{-17}	169.8
$[MQ+H]^+$-a6	40.5	90.7	6.14533×10^{-17}	166.7
$[MQ+H]^+$-b1	11.9	2.3	0.188965404	180.6
$[MQ+H]^+$-b2	28.7	18.3	0.000297272	174.5
$[MQ+H]^+$-b3	9.0	0.0	0.477910296	178.4
$[MQ+H]^+$-b4	0.0	0.9	0.332402888	179.6
$[MQ+H]^+$-b5	17.1	18.0	0.000335517	179.7
$[MQ+H]^+$-b6	21.9	21.3	8.86234×10^{-5}	174.4

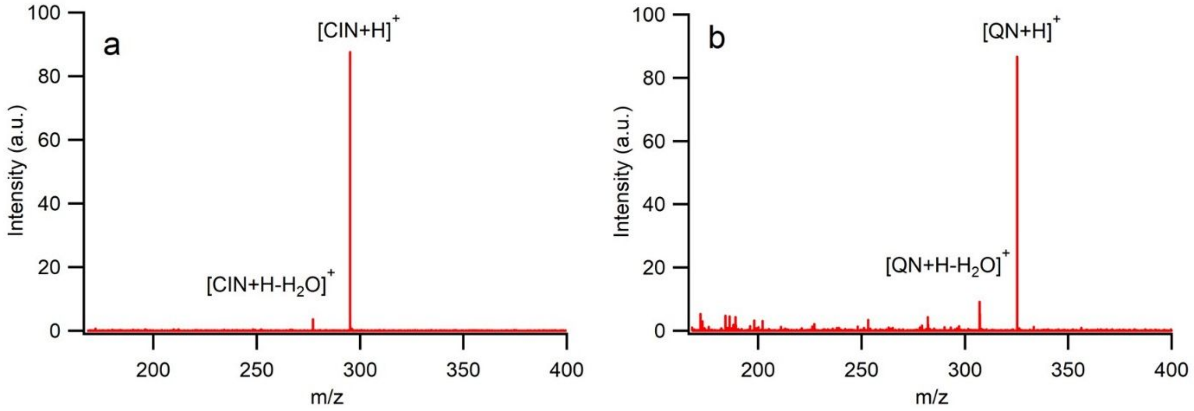

Figure S21. APCI-mass spectra of (a) CIN and (b) QN with CID voltages of 10 and 20 V, respectively.

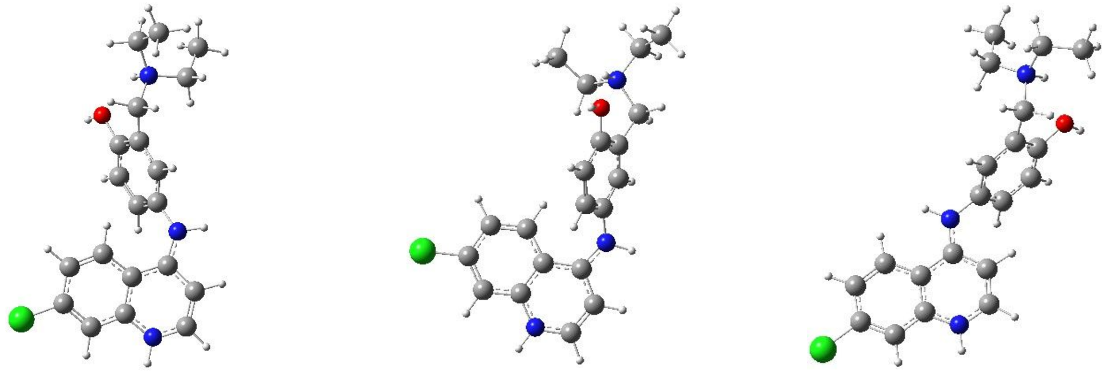

[AQ+2H]$^{2+}$-a **[AQ+2H]$^{2+}$-b** **[AQ+2H]$^{2+}$-c**

Figure S22. Optimized structures of di-protonated forms of Amodiaquine, [AQ+2H]$^{2+}$, in the gas phase.

Table S11. The calculated relative Gibbs free energies of different conformers of [AQ+2H]$^{2+}$ in the gas phase and in aqueous solution and their calculated CCS_{N2} values. (Experimental $^{DT}CCS_{N2}$ = 237.8 Å^2).

Ion	ΔG in gas phase (kJ mol^{-1})	ΔG in water (kJ mol^{-1})	CCS_{N2} (Å^2)
[AQ+2H]$^{2+}$-a	19.5	12.2	235.6
[AQ+2H]$^{2+}$-b	21.5	8.6	234.6
[AQ+2H]$^{2+}$-c	0.0	0.0	234.8

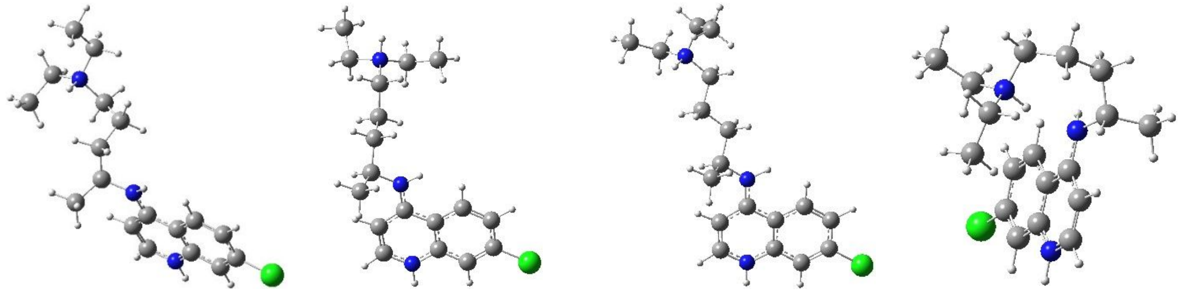

$[CQ+2H]^{2+}$-a $[CQ+2H]^{2+}$-b $[CQ+2H]^{2+}$-c $[CQ+2H]^{2+}$-d

Figure S23. Optimized structures of di-protonated forms of Chloroquine, $[CQ+2H]^{2+}$, in the gas phase.

Table S12. The calculated relative Gibbs free energies of different conformers of $[CQ+2H]^{2+}$ in the gas phase and in aqueous solution and their calculated CCS_{N2} values. (Experimental $^{DT}CCS_{N2}$ = 236.4 Å^2).

Ion	ΔG in gas phase (kJ mol^{-1})	ΔG in water (kJ mol^{-1})	CCS_{N2} (Å^2)
$[CQ+2H]^{2+}$-a	16.1	1.8	233.5
$[CQ+2H]^{2+}$-b	8.9	0.0	233.0
$[CQ+2H]^{2+}$-c	0.0	0.2	236.3
$[CQ+2H]^{2+}$-d	44.0	7.4	230.2

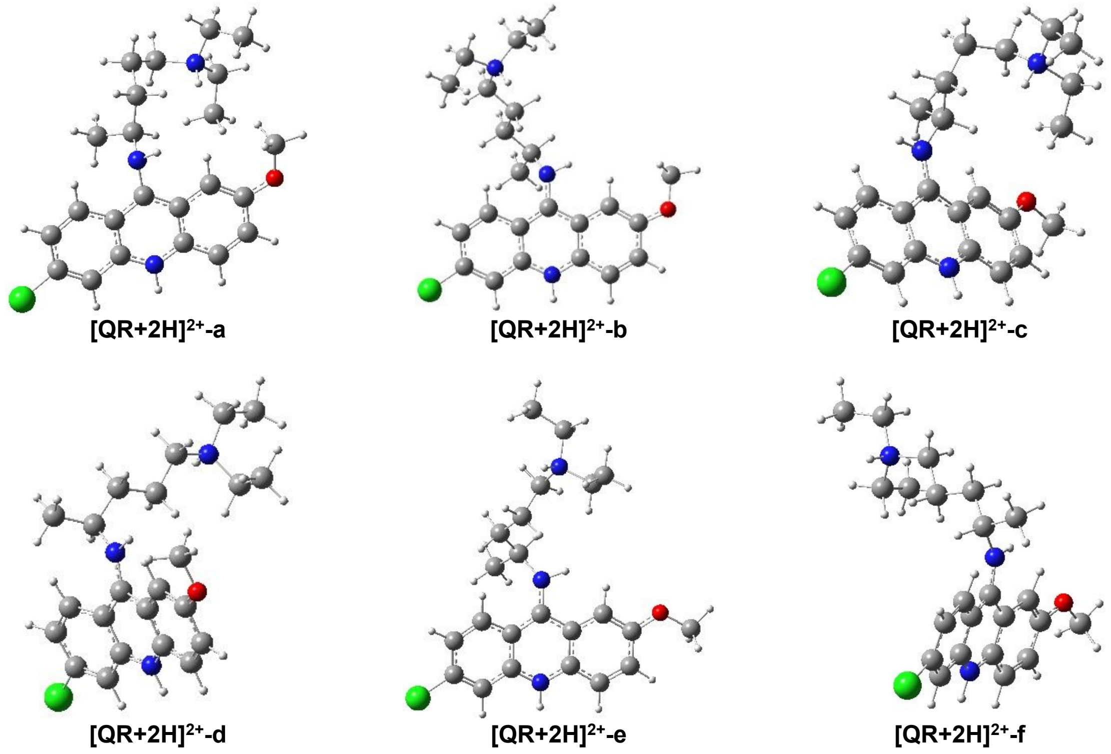

Figure S24. Optimized structures of di-protonated forms of Quinacrine, $[QR+2H]^{2+}$, in the gas phase.

Table S13. The calculated relative Gibbs free energies of different conformers of $[QR+2H]^{2+}$ in the gas phase and in aqueous solution and their calculated CCS_{N2} values. (Experimental $^{DT}CCS_{N2}$ = 252.8 Å^2).

Ion	ΔG in gas phase (kJ mol^{-1})	ΔG in water (kJ mol^{-1})	CCS_{N2} (Å^2)
$[QR+2H]^{2+}$-a	50.0	50.4	238.8
$[QR+2H]^{2+}$-b	19.0	25.2	247.8
$[QR+2H]^{2+}$-c	12.4	7.3	245.2
$[QR+2H]^{2+}$-d	3.6	3.5	245.5
$[QR+2H]^{2+}$-e	26.7	33.4	251.9
$[QR+2H]^{2+}$-f	0.0	0.0	248.5

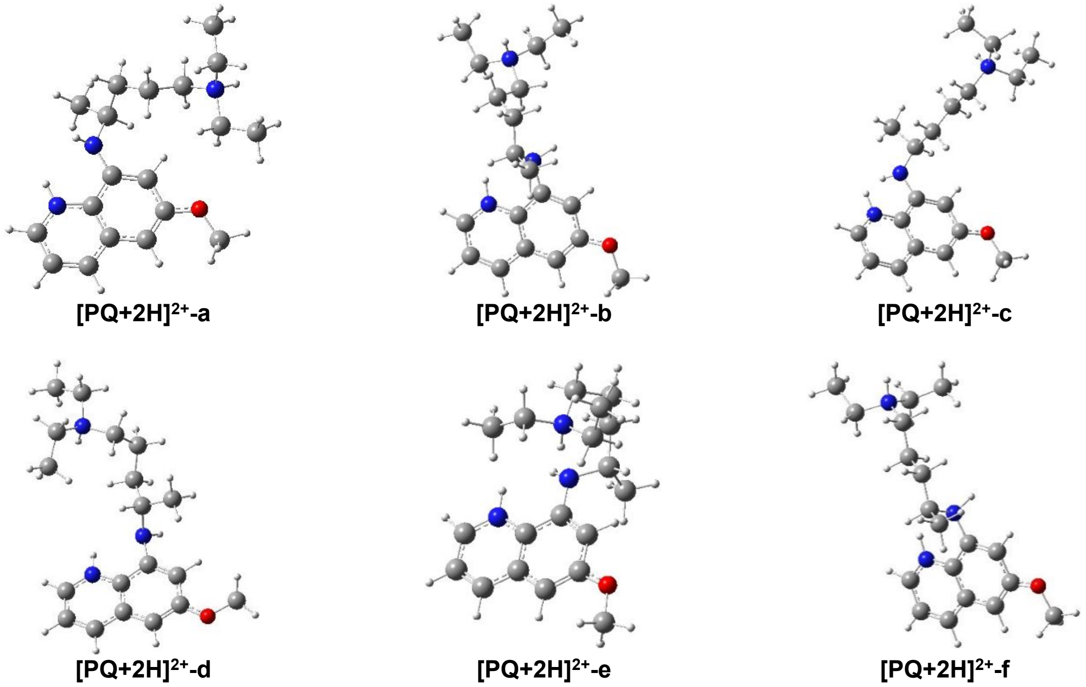

Figure S25. Optimized structures of di-protonated forms of Pamaquine, [PQ+2H]$^{2+}$, in the gas phase.

Table S14. The calculated relative Gibbs free energies of different conformers of [PQ+2H]$^{2+}$ in the gas phase and in aqueous solution and their calculated CCS_{N2} values. (Experimental $^{DT}CCS_{N2}$ = 234.1 Å^2).

Ion	ΔG in gas phase (kJ mol^{-1})	ΔG in water (kJ mol^{-1})	CCS$_{N2}$ (Å^2)
[PQ+2H]$^{2+}$-a	27.8	3.6	223.7
[PQ+2H]$^{2+}$-b	28.0	12.4	236.7
[PQ+2H]$^{2+}$-c	0.0	5.1	234.4
[PQ+2H]$^{2+}$-d	40.9	16.0	228.1
[PQ+2H]$^{2+}$-e	34.3	0.0	232.4
[PQ+2H]$^{2+}$-f	13.2	3.5	232.9

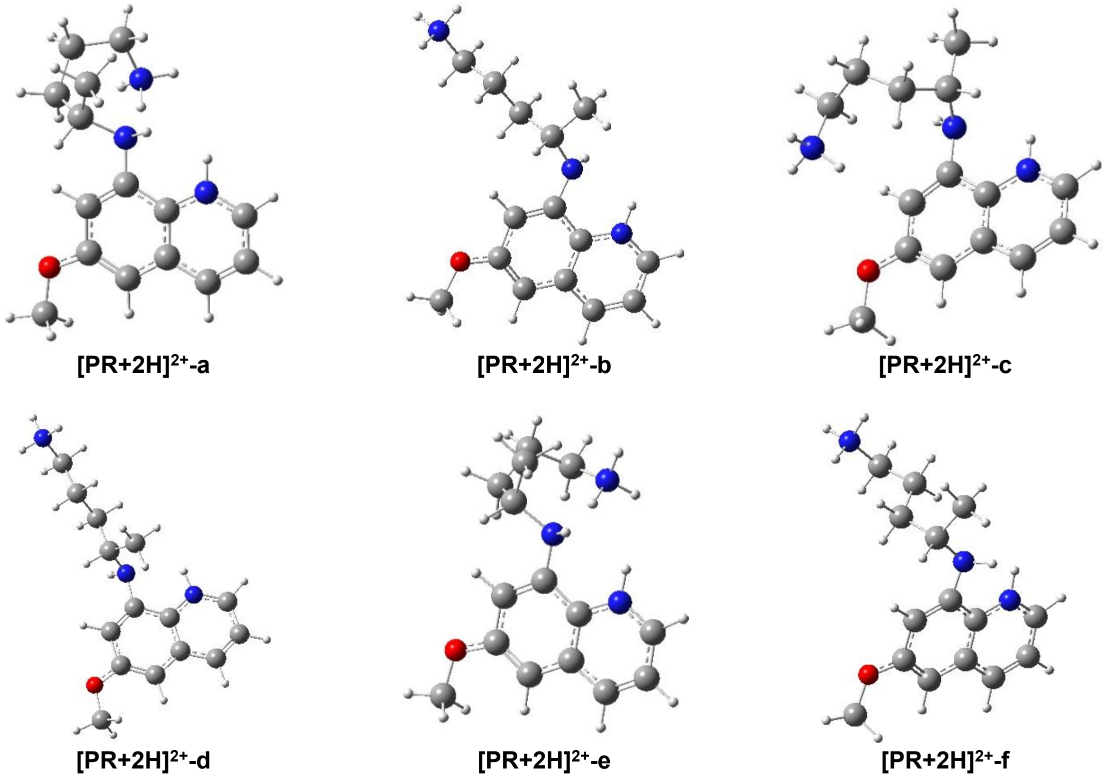

Figure S26. Optimized structures of di-protonated forms of Primaquine, $[PR+2H]^{2+}$, in the gas phase.

Table S15. The calculated relative Gibbs free energies of different conformers of $[PR+2H]^{2+}$ in the gas phase and in aqueous solution and their calculated CCS_{N2} values.

Ion	ΔG in gas phase (kJ mol^{-1})	ΔG in water (kJ mol^{-1})	CCS_{N2} ($Å^2$)
$[PR+2H]^{2+}$-a	44.2	5.4	222.5
$[PR+2H]^{2+}$-b	9.8	0.0	227.4
$[PR+2H]^{2+}$-c	49.6	23.2	221.4
$[PR+2H]^{2+}$-d	0.0	0.7	227.1
$[PR+2H]^{2+}$-e	39.9	4.7	221.1
$[PR+2H]^{2+}$-f	15.7	0.4	227.2

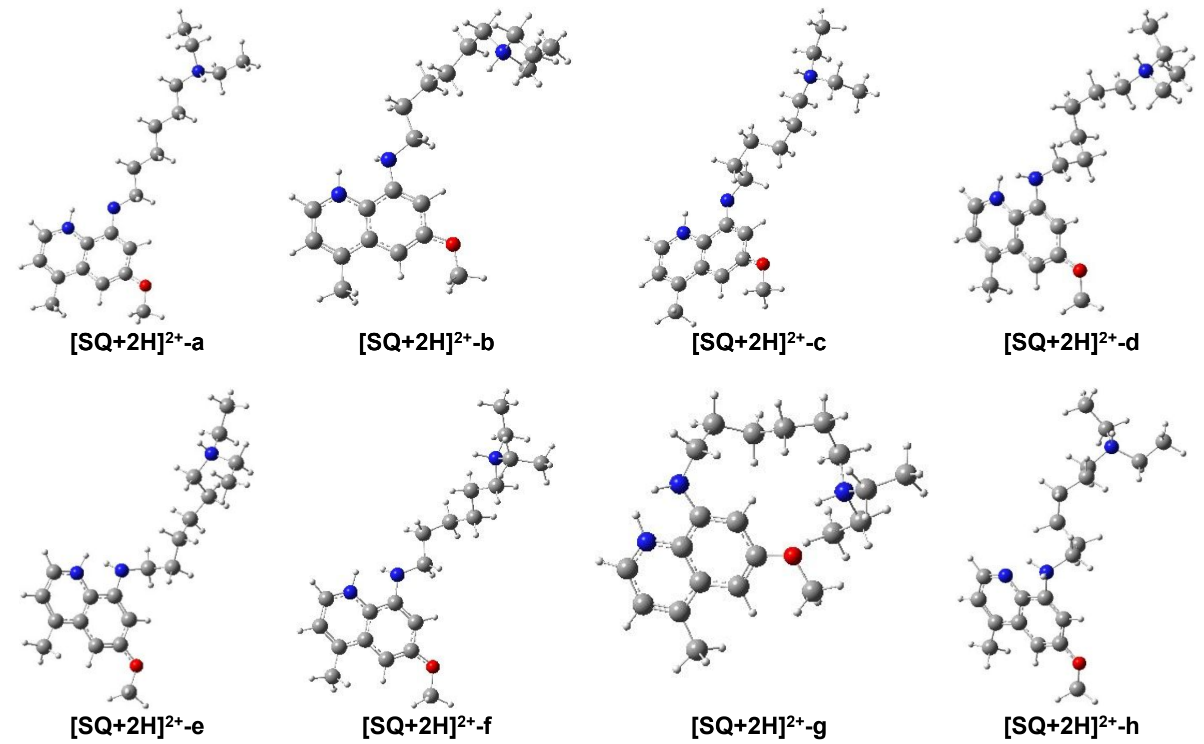

Figure S27. Optimized structures of di-protonated forms of Sitamaquine, [SQ+2H]$^{2+}$, in the gas phase.

Table S16. The calculated relative Gibbs free energies of different conformers of [SQ+2H]$^{2+}$ in the gas phase and in aqueous solution and their calculated CCS_{N2} values. (Experimental $^{DT}CCS_{N2}$ = 243.0 Å^2).

Ion	ΔG in gas phase (kJ mol^{-1})	ΔG in water (kJ mol^{-1})	CCS_{N2} (Å^2)
[SQ+2H]$^{2+}$-a	0.0	7.7	240.6
[SQ+2H]$^{2+}$-b	17.0	5.5	239.5
[SQ+2H]$^{2+}$-c	12.7	11.3	241.4
[SQ+2H]$^{2+}$-d	20.6	0.0	244.3
[SQ+2H]$^{2+}$-e	31.2	11.1	239.1
[SQ+2H]$^{2+}$-f	17.8	13.7	240.8
[SQ+2H]$^{2+}$-g	46.4	6.5	226.2
[SQ+2H]$^{2+}$-h	58.3	25.4	244.0

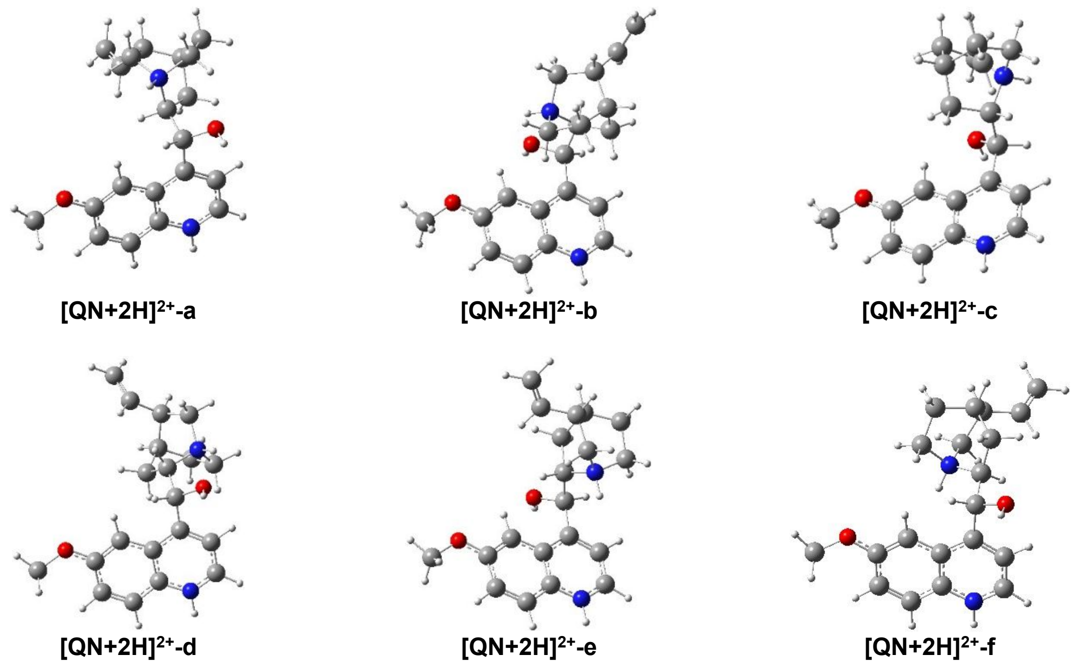

Figure S28. Optimized structures of di-protonated forms of Quinine, $[QN+2H]^{2+}$, in the gas phase.

Table S17. The calculated relative Gibbs free energies of different conformers of $[AQ+2H]^{2+}$ in the gas phase and in aqueous solution and their calculated CCS_{N2} values. (Experimental $^{DT}CCS_{N2}$ = 231.2 Å^2).

Ion	ΔG in gas phase (kJ mol^{-1})	ΔG in water (kJ mol^{-1})	CCS_{N2} (Å^2)
$[QN+2H]^{2+}$-a	0.0	0.0	228.7
$[QN+2H]^{2+}$-b	16.7	16.9	228.4
$[QN+2H]^{2+}$-c	13.9	12.9	231.1
$[QN+2H]^{2+}$-d	4.9	8.6	229.1
$[QN+2H]^{2+}$-e	23.0	14.1	230.6
$[QN+2H]^{2+}$-f	18.9	13.7	229.9

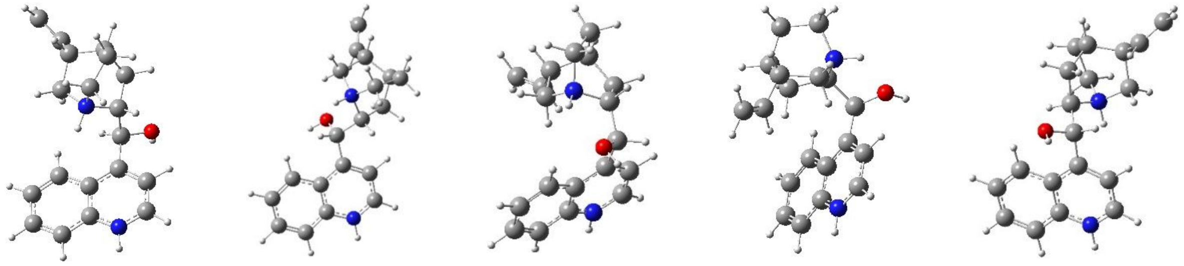

Figure S29. Optimized structures of di-protonated forms of Cinchonine, $[CIN+2H]^{2+}$, in the gas phase.

Table S18. The calculated relative Gibbs free energies of different conformers of $[CIN+2H]^{2+}$ in the gas phase and in aqueous solution and their calculated CCS_{N2} values. (Experimental $^{DT}CCS_{N2}$ = 229.6 Å^2).

Ion	ΔG in gas phase (kJ mol^{-1})	ΔG in water (kJ mol^{-1})	CCS_{N2} (Å^2)
$[CIN+2H]^{2+}$-a	18.9	6.9	225.0
$[CIN+2H]^{2+}$-b	16.7	17.3	221.3
$[CIN+2H]^{2+}$-c	11.1	10.5	221.9
$[CIN+2H]^{2+}$-d	0.0	0.0	224.3
$[CIN+2H]^{2+}$-e	20.0	7.6	227.0

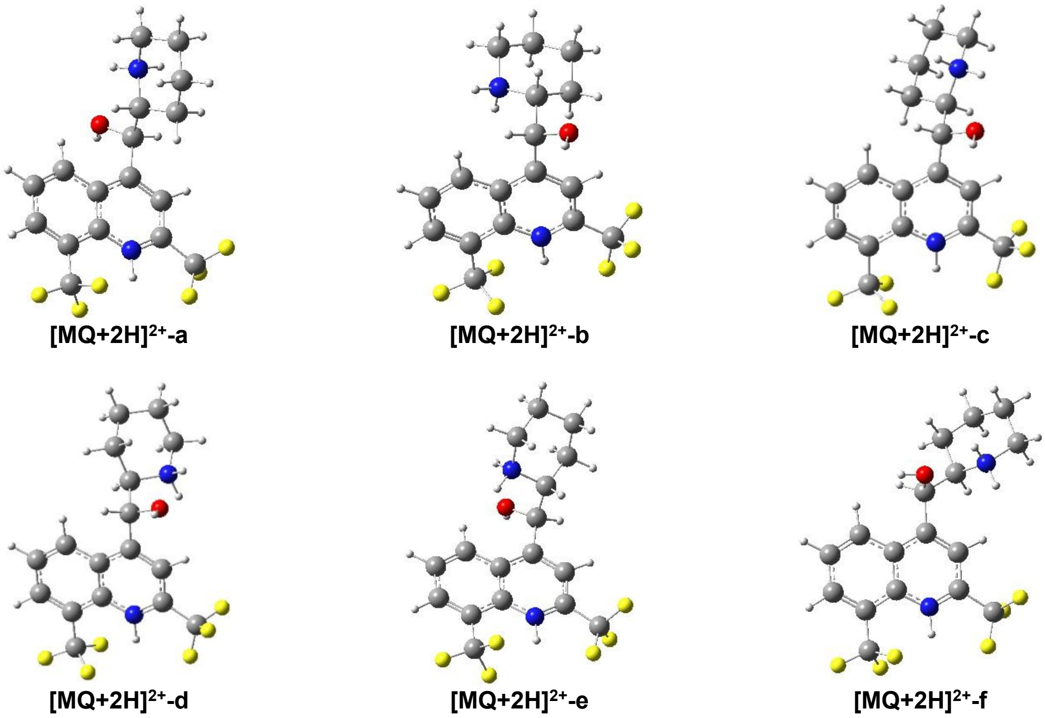

Figure S30. Optimized structures of di-protonated forms of Mefloquine, $[MQ+2H]^{2+}$, in the gas phase.

Table S19. The calculated relative Gibbs free energies of different conformers of $[MQ+2H]^{2+}$ in the gas phase and in aqueous solution and their calculated CCS_{N2} values.

Ion	ΔG in gas phase (kJ mol^{-1})	ΔG in water (kJ mol^{-1})	CCS_{N2} (Å^2)
$[MQ+2H]^{2+}$-a	3.8	5.8	229.0
$[MQ+2H]^{2+}$-b	41.6	28.5	230.4
$[MQ+2H]^{2+}$-c	0.0	0.0	228.6
$[MQ+2H]^{2+}$-d	9.8	8.1	228.8
$[MQ+2H]^{2+}$-e	24.7	20.4	231.3
$[MQ+2H]^{2+}$-f	28.3	30.2	227.9

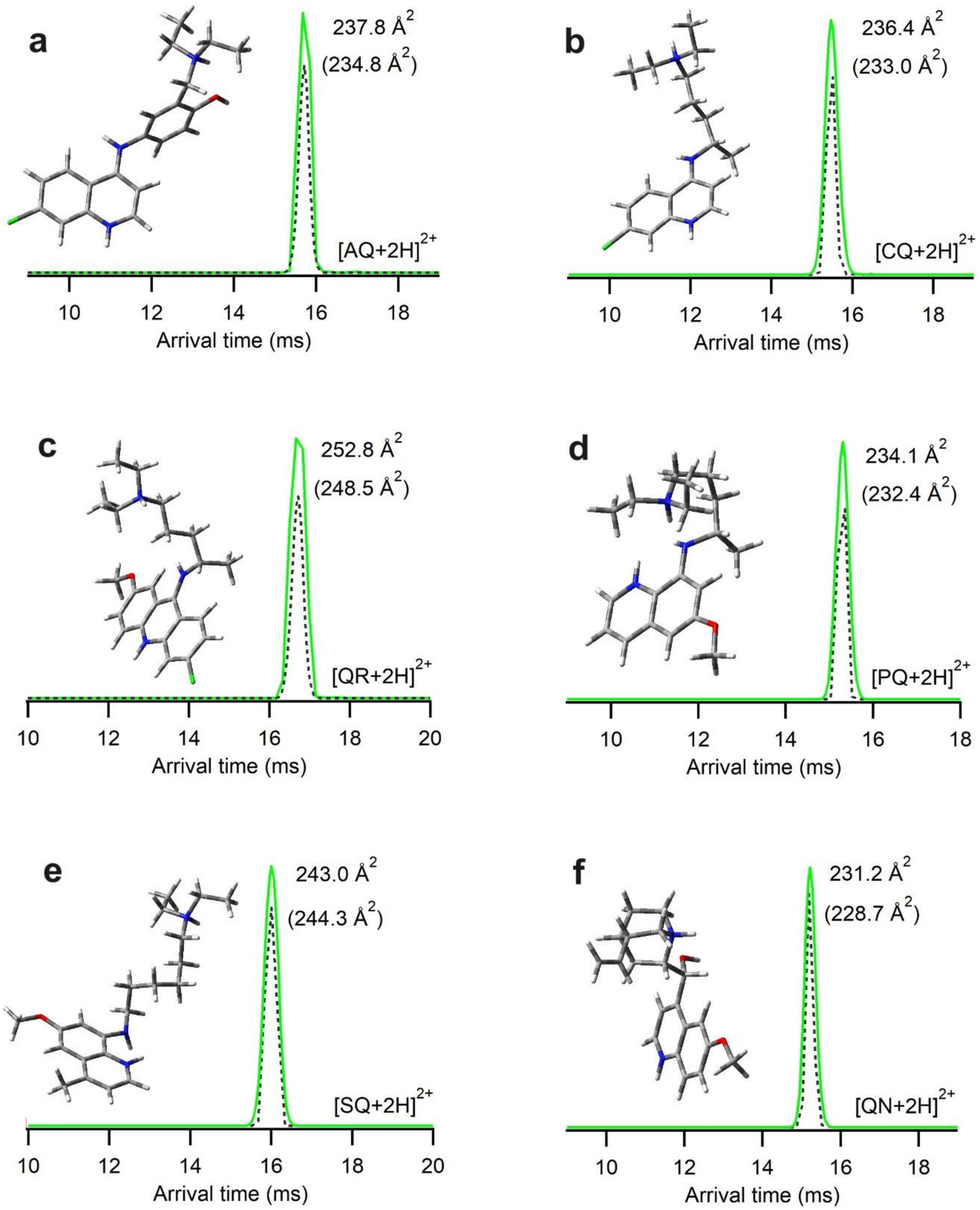

Figure S31. The ion mobility spectra and the optimized structures the most stable conformers of (a) $[AQ+2H]^{2+}$, (b) $[CQ+2H]^{2+}$, (c) $[QR+2H]^{2+}$, (d) $[PQ+2H]^{2+}$, (e) $[SQ+2H]^{2+}$, and (f) $[QN+2H]^{2+}$. The numbers in parenthesis are the theoretical calculated CCS_{N2} values for the shown structures.

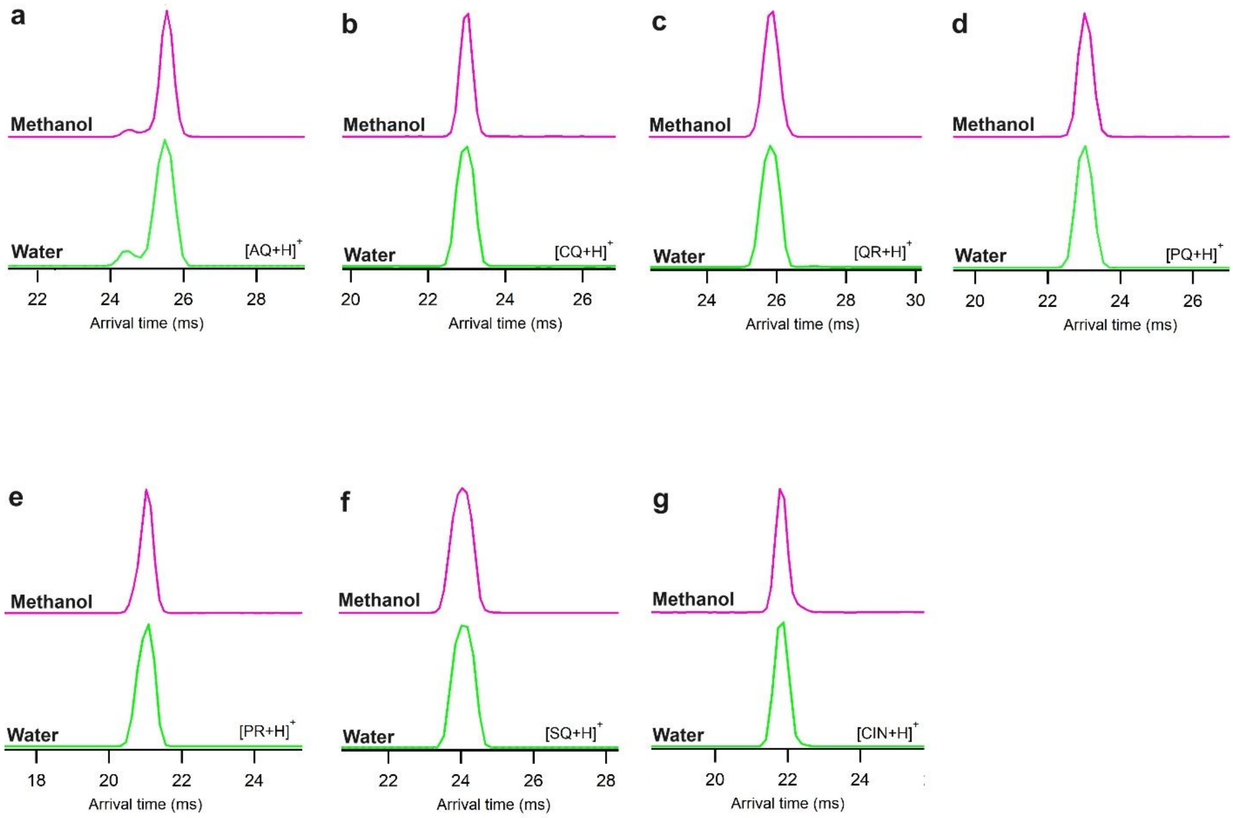

Figure S32. Comparison of the ESI-ion mobility spectra of (a) $[AQ+H]^+$, (b) $[CQ+H]^+$, (c) $[QR+H]^+$, (d) $[PQ+H]^+$, (e) $[PR+H]^+$, (f) $[SQ+H]^+$, and (g) $[CIN+H]^+$ in water and methanol (CH_3OH:H_2O, 90:10 v/v) solvents.

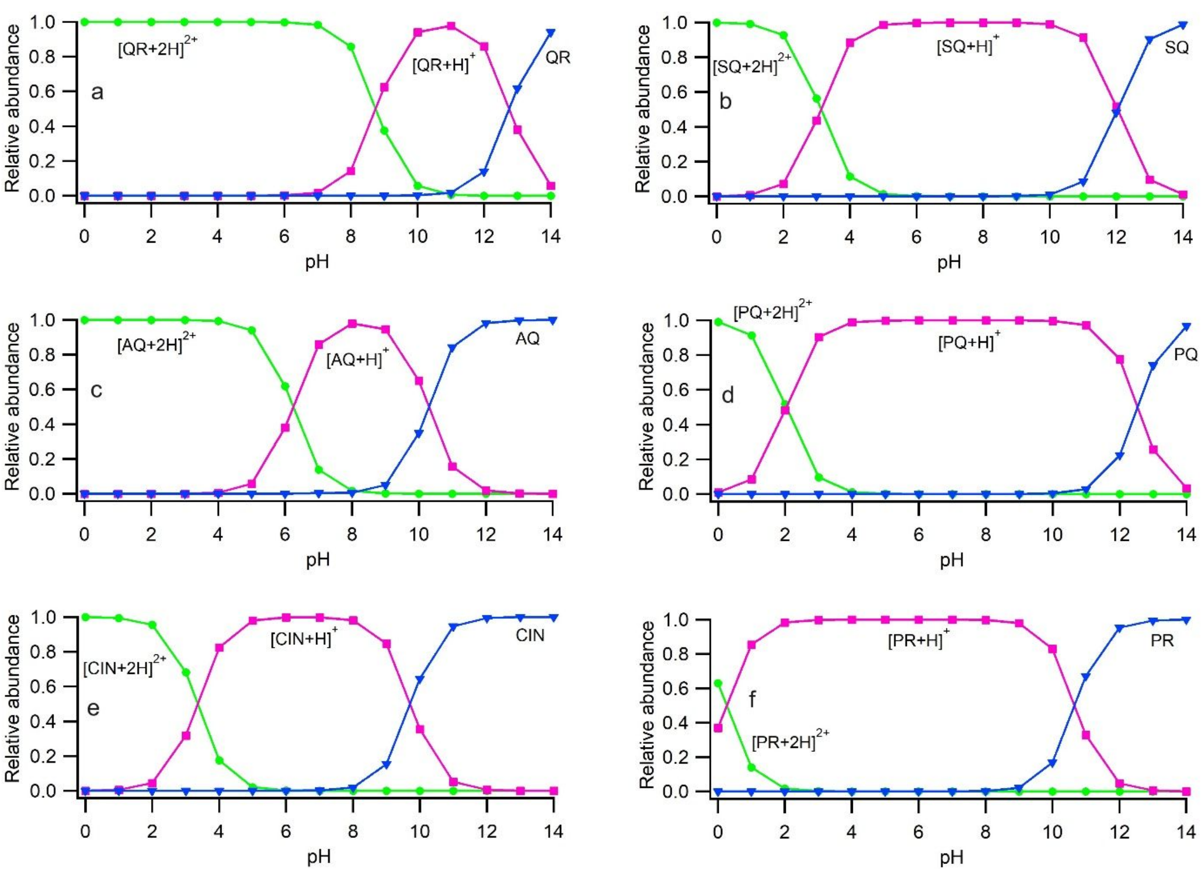

Figure S33. The calculated relative abundances of the $[M+2H]^{2+}$, $[M+H]^{+}$, and M species of (a) QR, (b) SQ, (c) AQ, (d) PQ, (e) CIN, and (f) PR at different pH in the aqueous solution.
